# High expression of vinculin predicts poor prognosis and distant metastasis and associates with influencing tumor-associated NK cell infiltration and epithelial-mesenchymal transition in gastric cancer

**DOI:** 10.18632/aging.202440

**Published:** 2021-02-01

**Authors:** Huafu Li, Chunming Wang, Linxiang Lan, Axel Behrens, Mona Tomaschko, Josue Ruiz, Qiao Su, Guangying Zhao, Cheng Yuan, Xing Xiao, Bo Li, Leping Yan, Wang Wu, Wuguo Li, Junzong Chen, Yulong He, Changhua Zhang

**Affiliations:** 1 Digestive Medicine Center, The Seventh Affiliated Hospital of Sun Yat-Sen University, Shenzhen, Guangdong, China; 2 Department of Gastrointestinal Surgery, The First Affiliated Hospital, Sun Yat-Sen University, Guangzhou, Guangdong, China; 3 Adult Stem Cell Laboratory, The Francis Crick Institute, London, UK; 4 Animal Experiment Center, The First Affiliated Hospital, Sun Yat-Sen University, Guangzhou, Guangdong, China; 5 Laboratory of Cancer Epigenome, Division of Medical Science, National Cancer Centre Singapore, Singapore; 6 Center of Scientific Research, The Seventh Affiliated Hospital of Sun Yat-sen University, Shenzhen, Guangdong, China

**Keywords:** vinculin, epithelial-mesenchymal transition, gastric cancer, NK cell Infiltration, DNA methylation

## Abstract

In the process of epithelial-mesenchymal transition (EMT), epithelial cancer cells transdifferentiate into mesenchymal-like cells with high motility and aggressiveness, resulting in the spread of tumor cells. Immune cells and inflammation in the tumor microenvironment are the driving factors of EMT, but few studies have explored the core targets of the interaction between EMT and tumor immune cells. We analyzed thousands of cases of gastric cancer and gastric tissue specimens of TCGA, CPTAC, GTEx and analyzing QPCR and IHC data of 56 gastric cancer patients in SYSU Gastric Cancer Research Center. It was known that EMT has an important connection with the infiltration of NK cells, and that the expression of vinculin may be the target of the phenomenon. The increased expression of vinculin is closely related to the aggressiveness and distant metastasis of cancer, which affects the survival prognosis of the patient. Moreover, through *in vitro* experiments under 3D conditions, we found that vinculin, cell invasion and metastasis are clearly linked. VCL can affect EMT and tumor immunity by regulating EPCAM gene expression. The role and mechanism of action of vinculin have been controversial, but this molecule may downregulate EpCAM (epithelial cellular adhesion molecule) and its own role in gastric cancer through DNA methylation, causing NK cells to enrich into tumor cells and kill tumor cells. At the same time, it promotes the occurrence of EMT, which in turn causes tumor metastasis and thus poorer prognosis.

## INTRODUCTION

Gastric cancer (GC) is one of the most aggressive malignant tumor in the world, and the third most common cause of cancer-related deaths due to its rapid progression into the late stage and high metastatic characteristics [1]. Despite advancements in diagnosis and systematic treatments, the prognosis for patients diagnosed with gastric cancer, especially metastatic gastric cancer, remains poor [2]. Epithelial-mesenchymal transition (EMT) is one of the key molecular steps in the process of distant metastasis of tumors. As the initial stage of metastasis, EMT is a complex process that includes not only the dissolution of cell junction, but also the loss of apico-basolateral polarity of epithelial cell when it transitions into mesenchymal cell [3]. Additionally, EMT confers stem cell-like properties on cancer cells, resistance to targeted therapy, and evasion to host immune surveillance [4]. Considering the extensive cellular and molecular changes that occur during the EMT process, the interaction between tumor cells and innate immune cells and adaptive immune cells in the tumor microenvironment may be altered. Several studies have shown that both innate immune cells and adaptive immune cells are driving factors for EMT [5]. Natural killer (NK) cells are innate lymphoid cells, known for their ability to recognize and quickly remove infected or transformed cells [6]. In humans, there is a correlation between low NK cell cytotoxicity in peripheral blood and increased cancer risk. On the contrary, NK cell infiltration of tumor tissue is related to better prognosis of patients with various malignant tumors, including gastric cancer [7]. However, research on the role of NK cells in tumor progression and metastasis is very limited. NK cells express MHC-independent inhibitory receptors, including the killer cell lectin-like receptor G1 (KLRG1). Type I epithelial cadherin (E-cad) gas been identified as an inhibitory ligand and is related to KLRG1 in both mice and humans [8]. Epithelial cell adhesion molecule (EpCAM) is considered to be a molecule that inhibits NK ligands. The high expression of EpCAM can promote NK cell toxicity by upregulating CEACAM1 [9]. However, presently there is not enough evidence to elucidate which pathway is responsible to regulate NK cells and EMT. For this reason, we grouped the pathways via EMT marker VIM, CDH1, S100A4, and EPCAM, then conducted GSEA and found that some of the pathways are immune-related, and discovered that NK cell infiltration is closely related to EMT. To this end, we conducted further research and found that vinculin may be the core target affecting NK cells and EMT.

## RESULTS

### EMT relative gene expression in GC will lead to distance metastasis and poor prognosis

**Figure 1:**
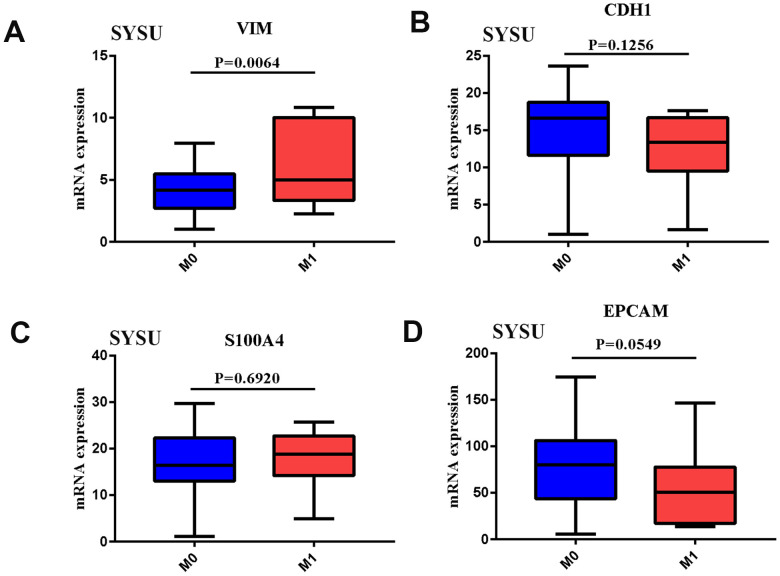
(**A**–**D**) VIM, CDH1, S100A4, EPCAM mRNA expression in GC tissues of SYSU Cohort patient of Distant metastases.

In order to clarify the role of EMT and distant metastasis, we first analyzed the mRNA expression of VIM, CDH1, S100A4 and EPCAM in human gastric cancer samples from the Cancer Genome Atlas (TCGA) data. The results showed that the expression of VIM was significantly increased in distantly metastatic tumor tissues (Supplementary Figure 1A), while the expression of the other three genes did not change significantly (Supplementary Figure 1B–1D). The expression level of VIM was also significantly up-regulated in the gastric cancer tissues of patients from our medical center (Figure 1A), while the expression levels of the other three genes did not change significantly (Figure 1B–1D). In addition, the relationship between EMT-related gene expression and survival prognosis in GC patients was also studied from TCGA and GEO databases. It was found that the high expression of VIM and the low expression of CDH1 and EPCAM were significantly related to the poor prognosis of patients (Supplementary Figure 2). It showed that EMT has a strong relationship with tumor metastasis and prognosis. In order to better identify gastric cancer patients with EMT, EMT and non-EMT patients were distinguished by combining the expression levels of VIM (high expression), CDH1 (low expression), S100A4 (high expression) and EPCAM (low expression). From Table 1, we can know that through the EMT marker, there were 56 EMT patients with high TCGA expression, and 289 EMT patients with low TCGA expression. Among them, there were more women with low EMT marker expression (P < 0.05), while cancer with high EMT marker expression were more aggressive (P < 0.05). Similarly, the four EMT makers were combined and used to distinguish the EMT status of 56 patients from our center, as shown in Supplementary Table 1. Patients with higher EMT had higher rate of distant metastasis and aggressiveness (P < 0.05). For gastric cancer patients, the mRNA expression data of 56 EMT and 289 non-EMT gastric cancer tissue samples were collected and summarized after preprocessing, then R package edge package was used to analyze the differential expression based on the log2 FC value of the top 50. A gene heat map was drawn, in which 50 mRNAs were up-regulated and 50mRNAs were downregulated. The specific genes were shown in Supplementary Figure 3. KEGG pathway analysis was used. The results showed that DEGs are mainly enriched in EMT-related pathways and immune-related cytokine pathways (see Supplementary Figure 4). It showed that EMT and immune-related pathways may be closely related. To this end, an EMT-grouped infiltrating immune cell analysis was conducted.

**Table 1:** Demographics of stomach carcinoma patients of TCGA.

	**High(56)**	**Low(289)**	**P**
male	26	197	<0.001
female	30	92	
Age	(67.58 ± 10.32)	(65.38 ± 11.11)	0.171
M staging			
M0	53	266	0.500
M1	3	23	
T staging			
T1	0	19	0.001
T2	1	63	
T3	31	128	
T4	13	79	
N staging			
N0	13	94	0.069
N1	23	70	
N2	9	64	
N3	11	60	
Differentiation			
High	3	7	0.126
Moderately	22	108	
Poorly	28	170	
Undifferentiation	3	4	

### NK cell inhibition may be an important factor that leads to EMT

**Figure 2:**
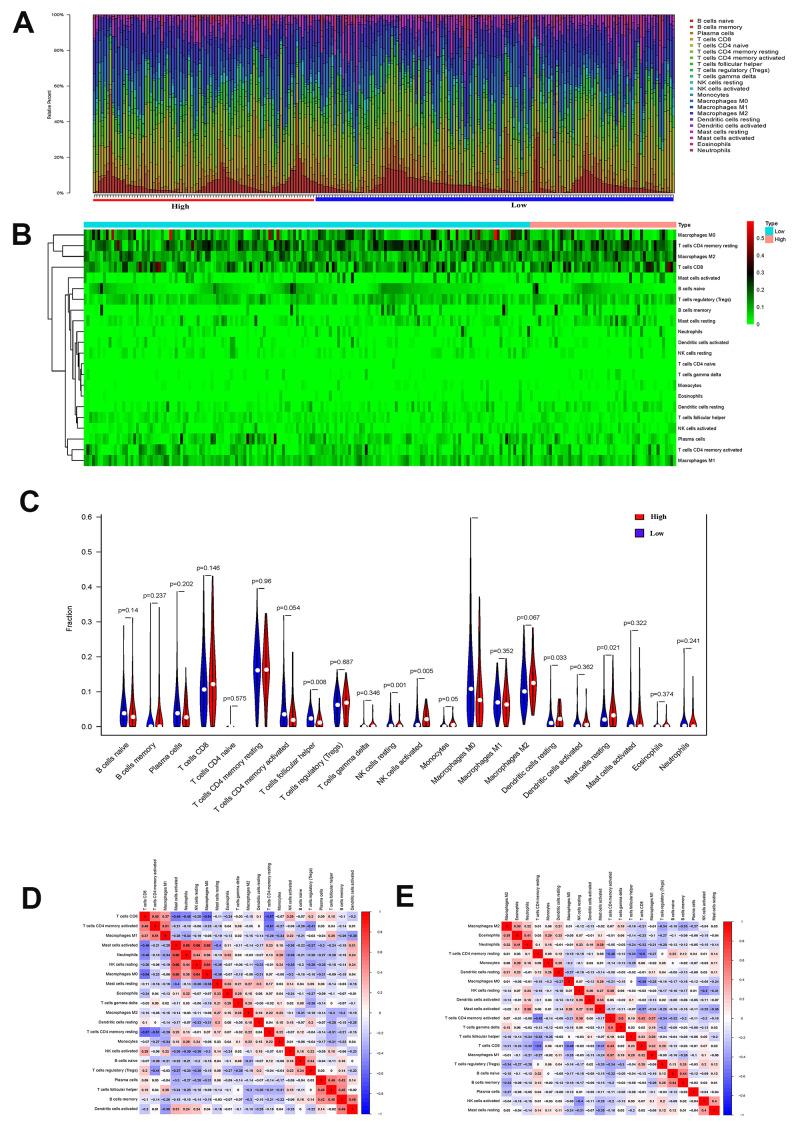
(**A**) The proportion of immune cell subsets in the EMT group and Non-EMT group. (**B**) Heat map of different immune cell subsets in EMT group and Non-EMT group. (**C**) The violin plot of the statistical differences between the tumor cells of different EMT groups. (**D**) The heat map of the correlation between tumor immune-infiltrating cells of EMT group. (**E**) Summary of the correlation between tumor immune-infiltrating cells of non-EMT group.

By comparing the proportions of immune cell subgroups between EMT groups, it is clear that the proportions of activated NK cells, resting dendritic cells, and resting mast cells in the EMT group were significantly higher than those in the non-EMT group (P<0.05). The proportion of T follicular helper cells, resting NK cells and M0 macrophages in the EMT group was significantly higher than that in the low expression group (P<0.05) (see Figure 2A–2C). In order to explore the correlation between different mutation groups of gastric tumor-infiltrating immune cells, the correlation between tumor-infiltrating immune cells in different EMT groups were compared. From Figure 2E, the correlation between tumor-infiltrating immune cells in different EMT groups is significantly different. For the EMT group, activated NK cell and resting NK cell were negatively correlated (Figure 2E). For the non-EMT group, T follicular helper cells and M0 macrophages were positively correlated. It showed that the activation of NK cells had an important connection with EMT.

### Vinculin may be an important factor affecting the inhibition of EMT and NK cells

Using the package ‘flashClust’ of WCGNA, cluster analysis was performed on 5,000 genes (Supplementary Figure 5A). The selection of soft threshold power is an important step in constructing WCGNA. The network topology structure with 1-20 threshold weights was analyzed, and the relatively balanced scale independence and average connectivity of WGCNA were determined. As shown in Supplementary Figure 5B and Supplementary Figure 5C, a power value of 4 was selected as the lowest power (0.9) of the scale-free topology ft index to generate a hierarchical cluster tree (dendrogram) of 5,000 genes. MEDissThres was set to 0.25 to merge similar modules (Supplementary Figure 5D), resulting in 58 modules (Supplementary Figure 5E). The gene statistics in each module were shown in Supplementary Table 2. The genes that cannot be included in any module were placed into the gray module and removed in the subsequent analyses. The interaction between 58 modules was analyzed and plotted into a network heat map (Supplementary Figure 5F). The results showed that each module was verified to be mutually independent and had relatively high independence, and the gene expression of each module is relatively independent as well. In addition, the feature genes were calculated and clustered according to their correlation to explore the co-expression similarity of all modules (Supplementary Figure 5G). As a result, these 58 modules were found to be mainly divided into two clusters. The heat map drawn from the adjacency relationship showed similar results (Supplementary Figure 5H). Turquoise was positively correlated with NK cell. Supplementary Figure 5I, 5J showed the relationship between the number of module members and GS in Turquoise.

**Figure 3:**
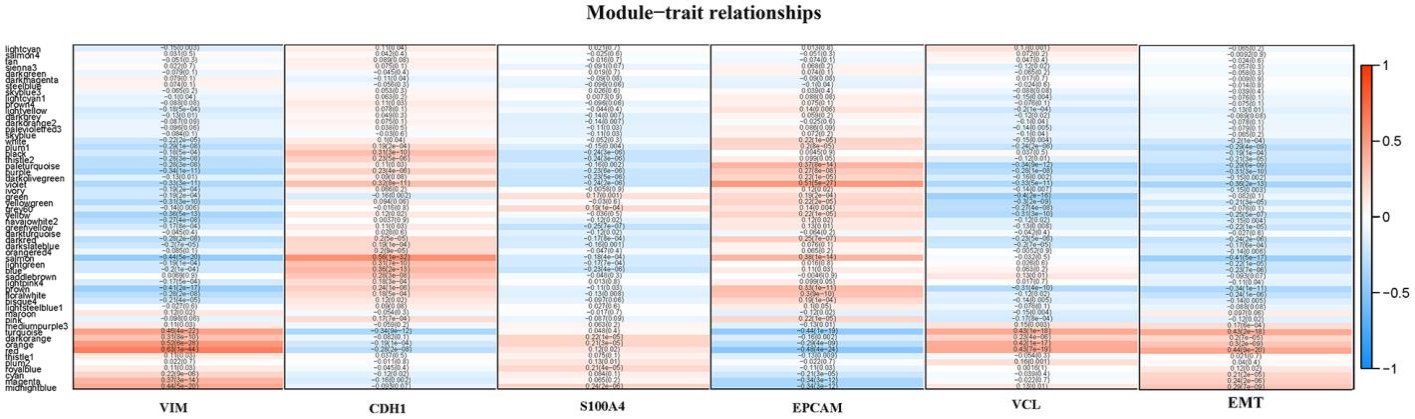
**A heat map of the correlation between module eigengenes and the status of VIM, CDH1, S100A4, EPCAM, VCL expression and EMT.**

The gene set of the Turquoise module was submitted to the protein-protein interaction analysis in STRING software, and the binding confidence interval of the cutoff value was set to 0.4. In the plugin Molecular Complex Detection (MCODE), the significant models with strong protein-protein connection were calculated and selected, with the default parameters (degree cut ≥ 2, node score cut ≥ 2, K-core ≥ 2, maximum depth = 100). P < 0.05 was considered statistically significant. By sorting the candidate genes in node degree, the core genes were selected for further analysis. Supplementary Figure 6A, 6B showed the hub gene in the Turquoise module, and within, VCL played a central role, showing that VCL was the core gene that caused NK cell infiltration. In order to study the specific role of VCL, GSEA method was used to analyze the pathways enriched by high expression and low expression of VCL. As seen in Supplementary Figure 7, VCL was not only related to immune-related pathways, but also closely related to EMT-related pathways. In order to clearly illustrate the correlation between VCL, VIM, CDH1, S100A4, and EPCAM and EMT, another WGCNA was run. The interaction between 57 modules was analyzed and a network heat map was plotted (Figure 3). The specifics of the WGCNA can be found in Supplementary Figure 8 and Supplementary Table 3. Among them, Red and EMT were positively correlated, indicating that the Red module has the strongest correlation with EMT. The genes of the Red module were analyzed (see Supplementary Figure 9) and results showed that the Red module was related to EMT-related signal pathways, further explaining the function of the Red module. It was also compatible with VIM (R = 0.63, P < 0.001), S100A4 (R = 0.12, P = 0.02), VCL (R = 0.43, P < 0.001). It was negatively correlated with CDH1 (R=0.28, P < 0.001), and EPCAM (R=0.48, P<0.001). Supplementary Figure 10 illustrated the relationship between the number of module members in Red and GS. Through the use of WGCNA, the correlation between VCL and EMT was found to be relatively strong.

### Using the CPTAC proteomics data to verify the role of VCL

**Figure 4:**
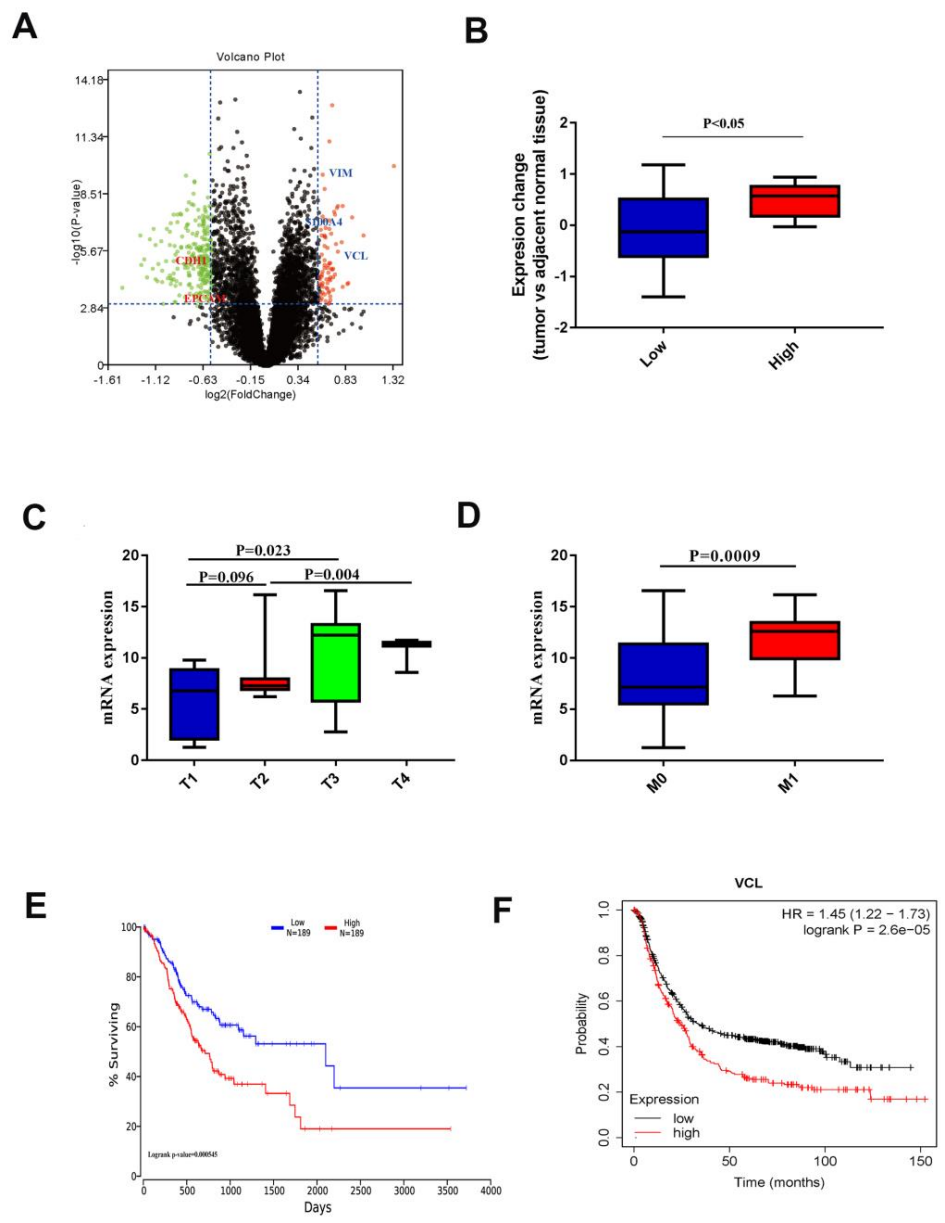
**(**A**) Volcano plot of different EMT histone protein expression. (**B**) Protein expression of VCL in different EMT groups. (**C**) VCL expression in different T stages. (**D**) VCL expression in different M stages. (**E**) Kaplan-Meier survival curves of overall survival in TCGA GC patients based on VCL mRNA expression. Log-rank test was used to compare differences between two groups. (**F**) Kaplan-Meier survival curves of overall survival in GEO GC patients based on VCL mRNA expression. Log-rank test was used to compare differences between two groups.**

Using the levels of EMT indicators, 19 EMT patients and 111 non-EMT patients were sorted into groups. Differential expression analysis was performed and a volcano plot was drawn through the t-test in R package. Among them, highly expressed VCL in the EMT group had statistical differences as shown in Figure 4A. By comparing the expression of VCL in EMT and non-EMT however (Figure 4B), the expression of VCL was significantly up-regulated in the EMT group.

### The VCL expression lead to distance metastasis and poor prognosis in gastric cancer

From the QPCR data of the 56 patients available in the Gastric Cancer Center of Sun Yat-sen University, the expression of VCL was proven to be significantly related to the cancer’s aggressiveness (T staging) (Figure 4C) and distant metastasis capabilities (Figure 4D). Based on the data of TCGA and GEO patients, 1,253 patients with gastric cancer were included for statistical analysis and the high expression of VCL was found to be significantly correlated with the prognosis of patients (Figure 4E, 4F). It could be said that the high expression of VCL may lead to enhanced invasion and distant metastasis of gastric cancer through EMT and NK cell infiltration, leading to poor prognosis of patients.

**Figure 5:**
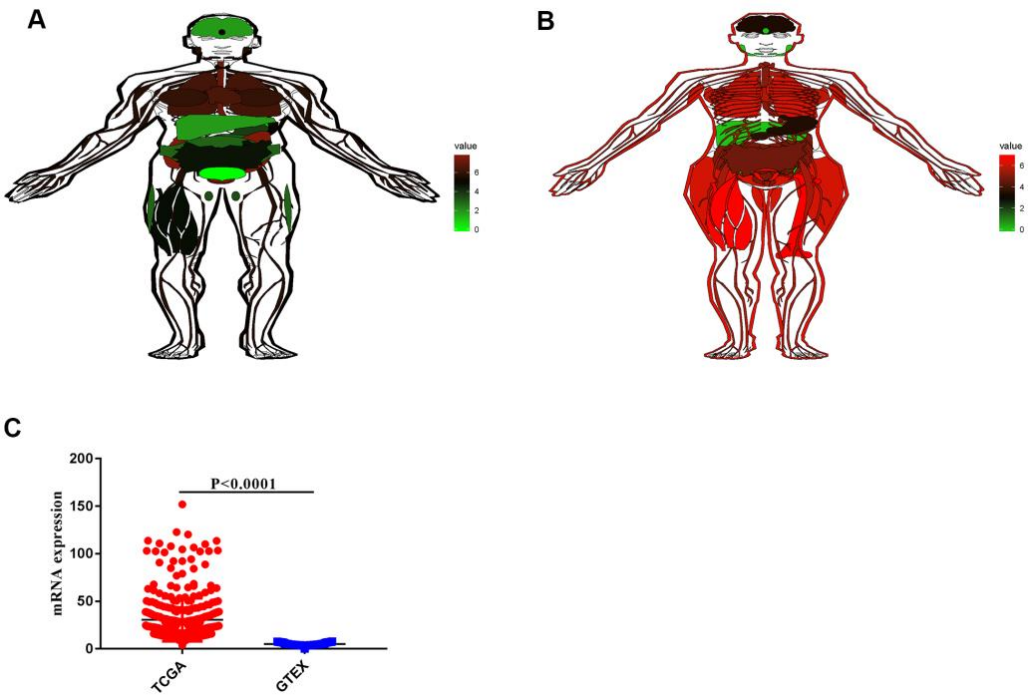
**RNA expression of VCL in normal tissues and tumors.** (**A**) VCL expression in different tissues of men. (**B**) VCL expression in different tissues of women.(**C**) Expression of VCL in tumor and normal tissues.

By analyzing the sequencing data of 9,783 normal tissue samples from the GTEx database, the expression profile of VCL in normal humans were mapped. The expression of VCL in males and females was different, but the expression of VCL was higher in the heart and lung tissues, but lower in the liver and stomach (see Figure 5A, 5B). By comparing the expression of VCL in gastric tissue samples from the TCGA gastric cancer database and the GTEx database, the expression of VCL in gastric cancer tissue was found to be significantly higher than that in normal gastric cancer tissue (see Figure 5C). It proved that VCL may have important significance in the occurrence and development of gastric cancer.

### VCL downregulation of EPCAM by CpG methylation

**Figure 6:**
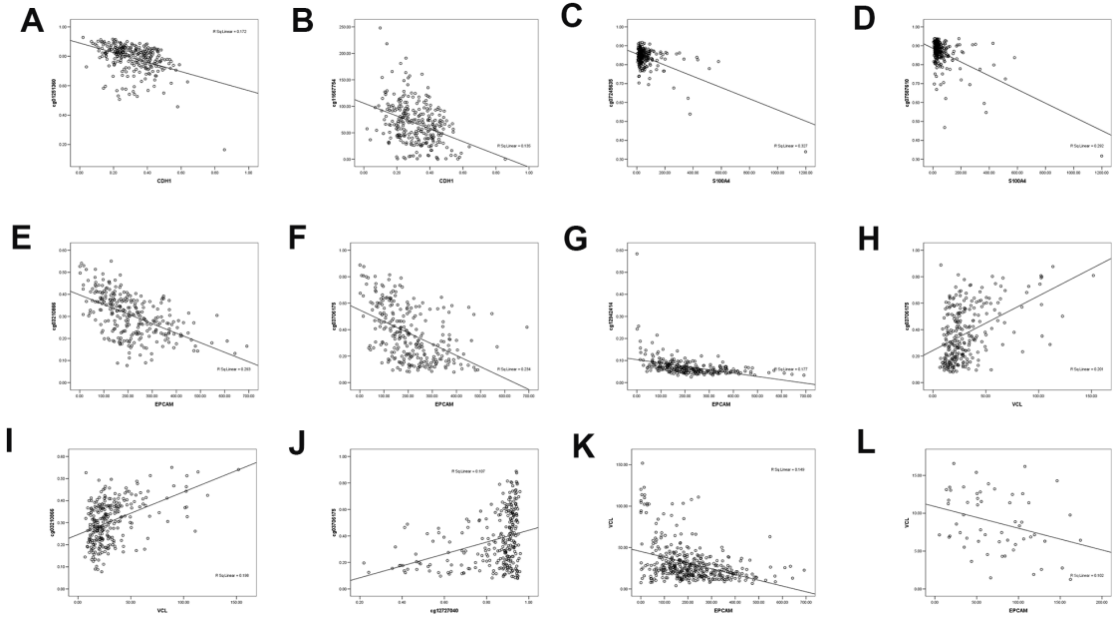
**The correlation between methylated sites and gene expression in gastric cancer patients.** (**A**) Negative linear correlation between CDH1 and CDH1 DNA methylation site cg01251360. (**B**) Negative linear correlation between CDH1 and CDH1 DNA methylation site cg11667754. (**C**) Negative linear correlation between S100A4 and S100A4 DNA methylation site cg07245635. (**D**) Negative linear correlation between S100A4 and S100A4 DNA methylation site cg07587610. (**E**) Negative linear correlation between EPCAM and EPCAM DNA methylation site cg03210866. (**F**) Negative linear correlation between EPCAM and EPCAM DNA methylation site cg03706175. (**G**) Negative linear correlation between EPCAM and EPCAM DNA methylation site cg12942414. (**H**) linear correlation between VCL and EPCAM DNA methylation site cg03706175. (**I**) linear correlation between CDH1 and EPCAM DNA methylation site cg03210866. (**J**) linear correlation between VCL DNA methylation site cg12727040 and EPCAM DNA methylation site cg03706175. (**K**) Negative linear correlation between VCL and EPCAM expression. (**L**) Negative linear correlation between VCL and EPCAM expression in SYSU.

The methylation of 7 CpG sites and the expression of quantitative trait loci (meQTLs) were evaluated in the genome. Since promoter hypermethylation played an important role in the inactivation of cancer-related genes, genes with low methylation and high expression levels were of particular interest [22]. By analyzing the DNA methylation data of TCGA gastric cancer database, there were 12 DNA methylation sites in VIM, 8 in CDH1, 3 in S100A4, 7 in EPCAM, and 6 in VCL. Linear analysis of DNA methylation and RNA expression showed that RNA expression of CDH1 was negatively correlated with its DNA methylation sites cg01251360 and cg11667754 (Figure 6A, 6B). The RNA expression of S100A4 was negatively correlated with its DNA methylation sites cg07245635 and cg07587610 (Figure 6C, 6D). The RNA expression of EPCAM was negatively correlated with its DNA methylation sites cg03210866, cg03706175 and cg012942414 (Figure 6E–6G). Through linear analysis of DNA methylation of VCL and RNA of EMT-related RNA, VCL RNA expression was found to be positively correlated with EPCAM DNA methylation (cg03706175, cg03210866) (Figure 6H, 6I). The DNA methylation site cg12727040 of VCL was positively correlated with the DNA methylation site cg0370617 of EPCAM (Figure 6J, 6K). And the expression level of VCL is negatively correlated with the expression level of EPCAM (Figure 6L), indicating that VCL may negatively regulate EPCAM expression through RNA expression and DNA methylation. This may be the main target of EMT caused by VCL.

### VCL downregulation affects GC cell line invasion and migration

**Figure 7:**
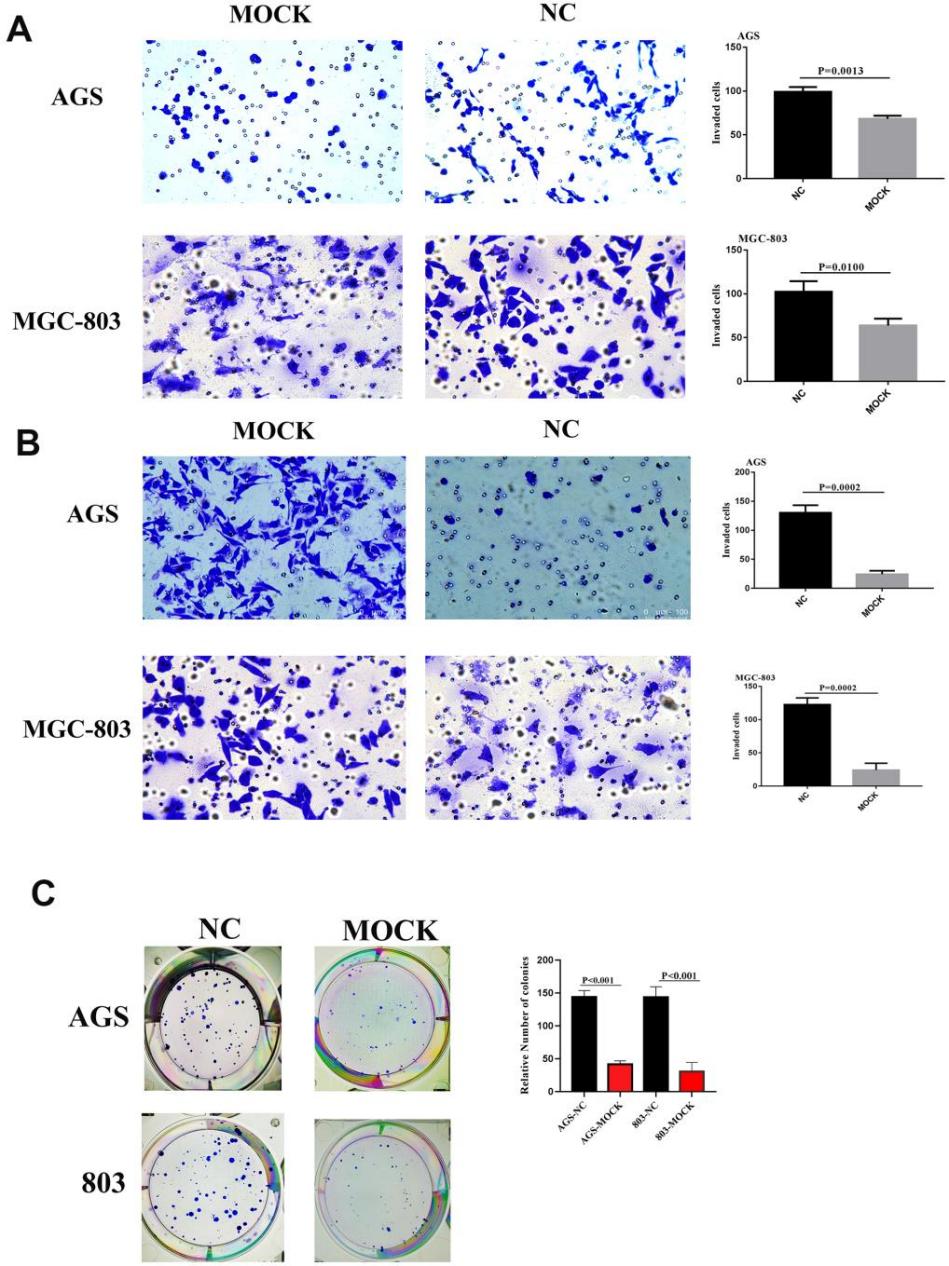
(**A**) Migrative ability of AGS and MGC-803 of VCL silent group and blank group. (**B**) Invasive ability of AGS and MGC-803 of VCL silent group and blank group. (**C**) Colony formation of AGS and MGC-803 of VCL silent group and blank group.

siRNA was used to reduce VCL levels in a group of genetically different GC cell lines (Supplementary Figure 10) to confirm its involvement in this aggressive phenomenon. In general, compared with the control group, downregulation of VCL strongly inhibited cell invasiveness (Figure 7A) and also reduced cell metastasis (Figure 7B). It showed that VCL had an important influence on the invasion and metastasis of gastric cancer.

Through colony formation assay, we found that the cell colony formation of the VCL knockout group was significantly less than that of the control group. It proved that VCL can promote the proliferation of tumor cells (Figure 7C).

### Immunohistochemical analysis showed that VCL expression was associated with EMT and distant metastasis in gastric cancer

**Figure 8:**
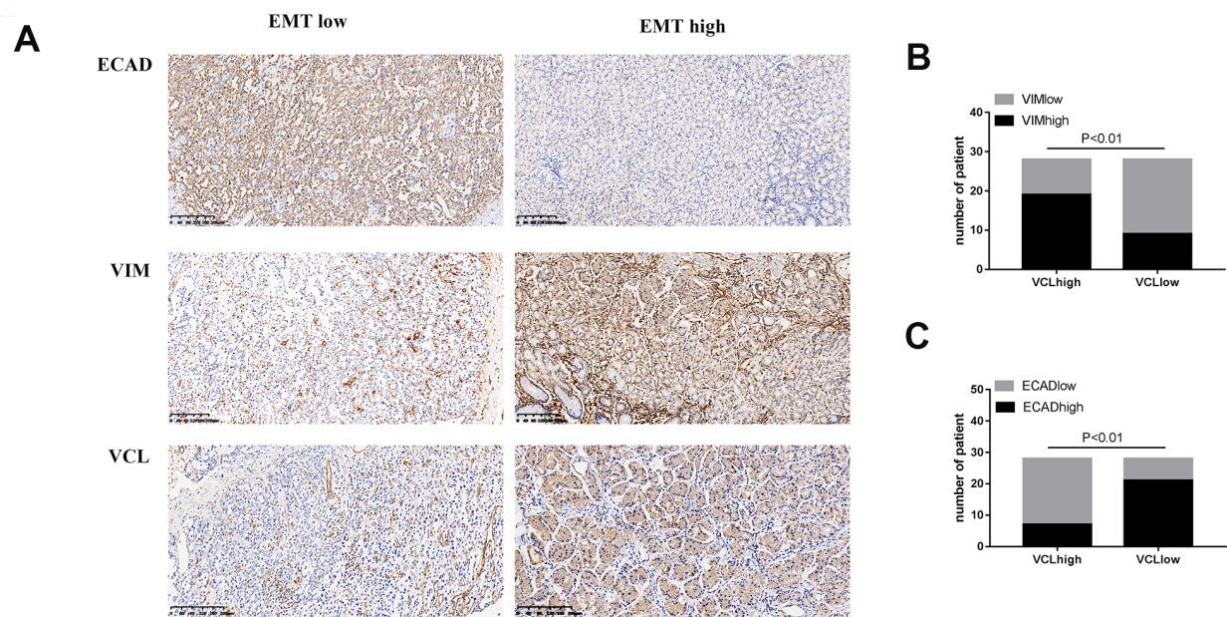
**The expression of VCL was positively correlated with EMT (**A**) IHC analysis of VIM, ECAD, and VCL in 56 human gastric cancer specimens (200X). (**B**) Correlation between VIM and VCL expression. (**C**) Correlation between ECAD and VCL expression.**

**Figure 9:**
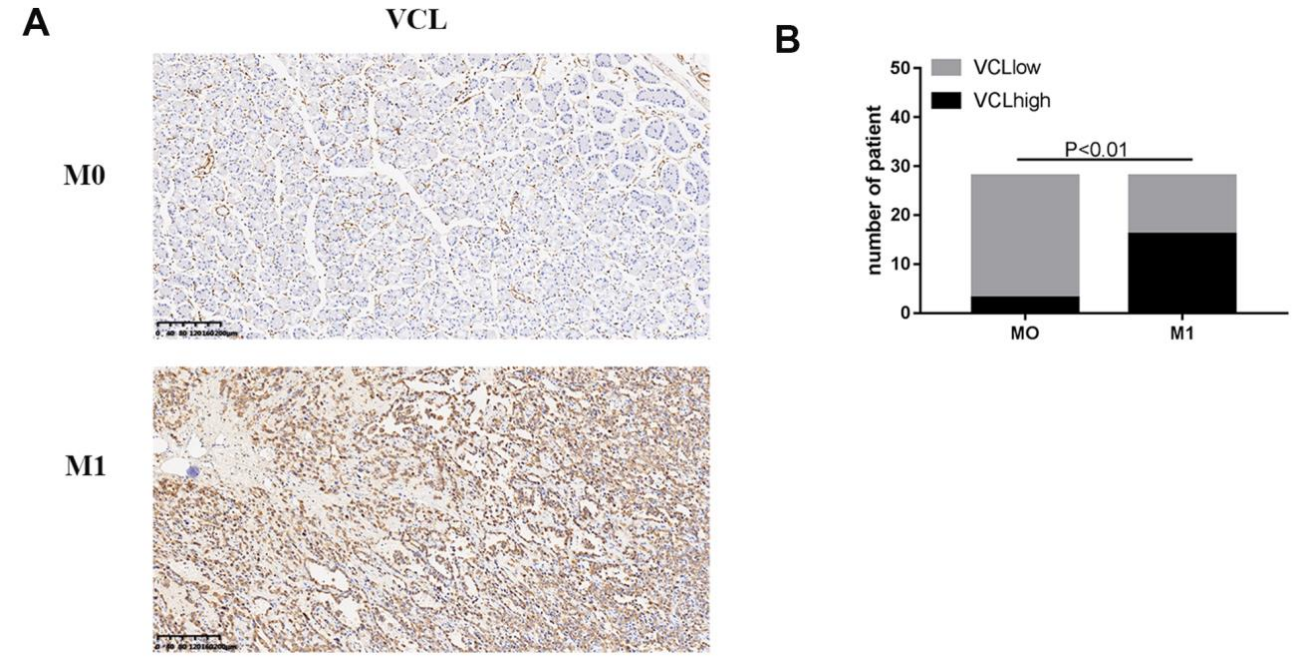
**The expression of VCL was positively correlated with M1 (**A**) IHC analysis of VCL in 56 human gastric cancer specimens (200X) of M0 and M1. (**B**) Correlation between M1 and VCL expression.**

The immunohistochemical status of 56 patients was analyzed and found that VCL expression was positively correlated with VIM and negatively correlated with ECAD, indicating that VCL had an important relationship with the development of EMT in gastric cancer (see Figure 8). Moreover, the expression of VCL was significantly correlated with distant metastasis of gastric cancer (see Figure 9).

### *In vivo* VCL may affect the occurrence of EMT by regulating the expression of EPCAM

**Figure 10:**
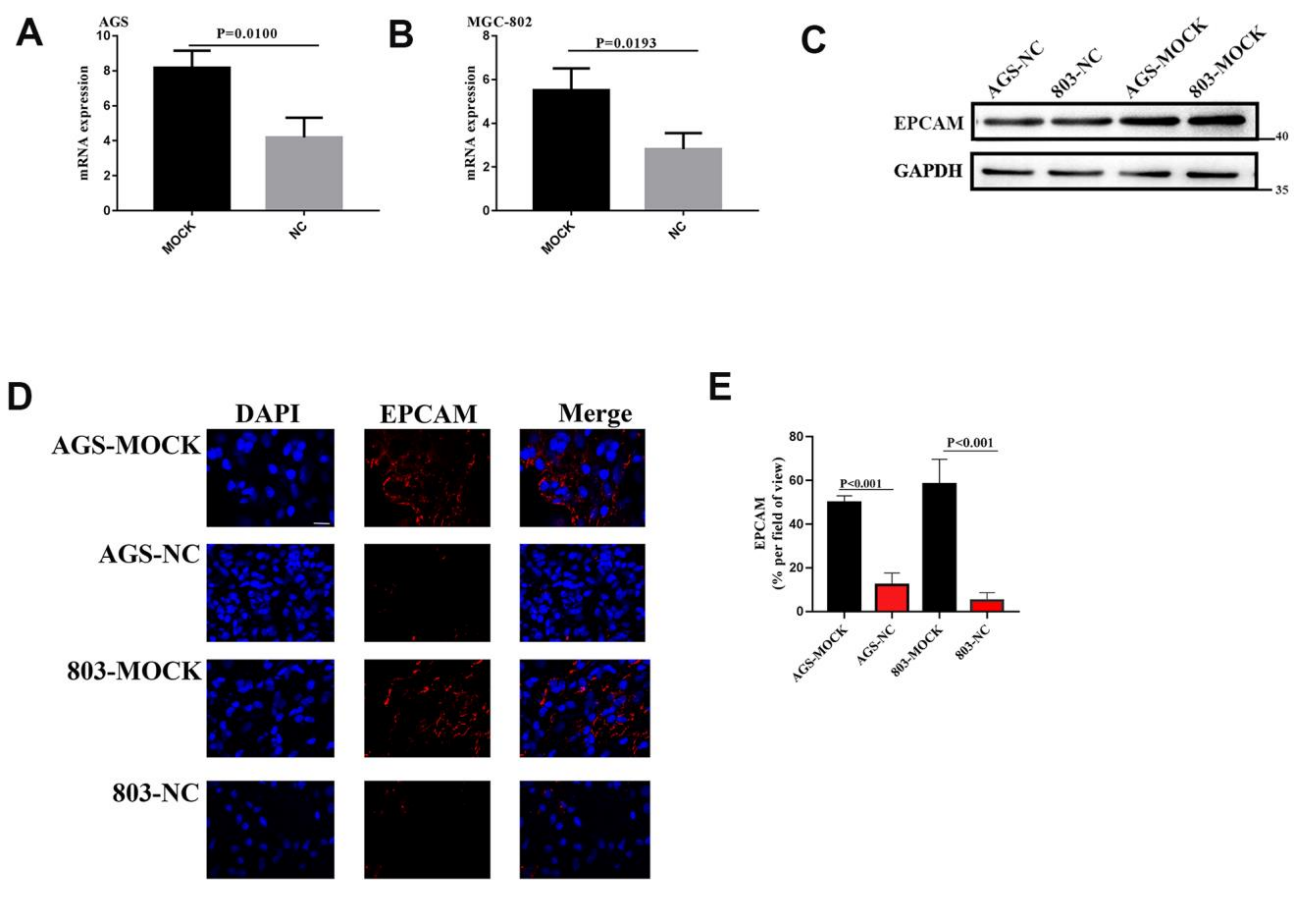
**(**A**, **B**) The EPCAM RNA expression of VCL silent group and blank group. (**C**) The EPCAM expression of VCL silent group and blank group. (**D**) Representative images of EPCAM (red) and DAPI (bule) formation in silent group and blank group cells. Scale bars, 10 mm. (**E**), proportion of EPCAM+ cells in tumor in (**D**).**

From above, we know that VCL regulated the expression of EPCAM through DNA methylation, and that EPCAM was an important target that affected EMT. In order to verify the relationship between VCL and EPCAM expression *in vivo*, the mRNA and total protein of the VCL knockdown group and the control group were extracted, and qPCR and protein electrophoresis were performed, results proved that the EPCAM expression of the VCL knockdown group was statistically significantly increased (Figure 10A–10C). Through immunofluorescence, we found that the expression of EPCAM in the VCL knockdown group was also significantly higher than that in the control group (Figure 10D, 10E). EPCAM was an adhesion molecule of epithelial cells. VCL could reduce the expression of EPCAM and thus reduce the adhesion between cells, thereby affecting cell migration.

## DISCUSSION

As one of the most significant features of malignant tumors, distant metastasis is a complex process affected by genetic and epigenetic modifications, accounting for more than 90% of cancer-related deaths [10]. EMT is of great significance to the occurrence and development of early tumor metastasis, and has since become a hot spot for research thus widely studied [11]. However, there are still few studies on EMT in gastric cancer. Gastric cancer is a common but very unique tumor. Finding the mechanism that leads to its distant metastasis is undoubtedly an important milestone for us in the battle to overcome its poor prognosis [12]. Natural killer (NK) cells are mainly large granular lymphocytes (LGL), accounting for 5-8% of human blood lymphocytes, and at least 70% of them have NK cell activity. These cells show natural cytotoxicity to a variety of human solid tumors, leukemia, and virus-infected target cells, but the target cells lack major histocompatibility complexes [13]. Studies had shown that the number of NK cells has an important impact on the prognosis of gastric cancer [7]. Interestingly, early studies had shown that EMT helps cancer cell escape T cell immune surveillance and allow them to leave the primary tumor site [14]. However, studies had also shown that simultaneous modulation of NK ligands due to EMT will cause the tumor cells to be vulnerable to NK-mediated cytotoxicity when they abandon the immunosuppressive primary tumor microenvironment, allowing for metastasis-specific control [5]. Our research found that while EMT can significantly recruit NK cells to tumors, it also led to metastasis. There is the deeper mechanism of EMT, which can simultaneously cause tumor metastasis and also lead to tumor immune-evasiveness [15]. And for this reason, looking into the relationship between EMT and NK cell may prove to be the gateway to a key target for future research.

Recent studies had shown that the important markers of EMT, namely vimentin, E-cadherin, FSP1 and EPCAM, that can effectively help us find tumor cells undergoing the EMT process [16]. While searching for the core genes of tumor-infiltrating NK cells through WGCNA, we found that VCL may be an important target that causes high expression of NK cells. And through VCL's GSEA and WGCNA, it was revealed that VCL is not only importantly related to NK cells, but also closely related to EMT. It showed that VCL may regulate NK cells and EMT through some way. In the past, vinculin was considered to be a tumor suppressor which inhibited tumor metastasis by supporting the growth of cells that rely on anchor connections and reducing cell viability [17]. However, the role of vinculin in regulating cell migration is far more complicated, with fundamental differences between 2D and 3D environments [18]. The influence of vinculin on the prognosis of gastric cancer has always been controversial. Chunhong Zhang et al. studied the expression of paxillin and vinculin in gastric cancer and precancerous lesions and their influence on the prognosis of gastric cancer, and found that the expression of vinculin was related to a better prognosis of gastric cancer patients [19]. However, Mingming Zhang et al. found that vinculin could promote the proliferation and migration of gastric cancer and predict a poor prognosis of patients with gastric cancer [20]. Thus, we need to solve this discrepancy through more thorough research. Through data of the QPCR of our gastric cancer patients and public database RNA sequence proteomics, we found that VCL was closely related to poor prognosis of patients and distant metastasis in them. *In vitro* 3D invasion and metastasis experiments proved that VCL can affect tumor metastasis in 3D. To verify the effect of vinculin on tumor cells, we had knocked down vinculin in gastric cancer cell lines *in vitro* to verify the effect of vinculin on tumors, and found that vinculin can play an important role by regulating the expression of EPCAM. It is known that EPCAM is an important target that affects EMT. Moreover, when Park et al. studied the regulatory effects of EPCAM on cytotoxicity of NK cell, they found that the high expression of EPCAM can cause tumor cells to escape the cytotoxic effects of NK cells by up-regulating CEACAM1 [9]. Chockley et al. found that EMT can recruit NK cells toward tumor cells, and may increase the cytotoxicity of NK cells and thus lead to tumor cell death [5]. Therefore, through our large amount of clinical data and TCGA and other database mining, we first confirmed that vinculin had an important influence on the migration and metastasis of gastric cancer. Through *in vivo* experiments, we have determined that VCL can affect EMT and tumor immunity by regulating the expression of EPCAM.

## CONCLUSIONS

For the first time, we have discovered that vinculin is the core gene regulating EMT and NK cells. It mediates the metastasis of tumor cells and brings about poor prognosis for patients by regulating EPCAM and affecting the enrichment of NK cells. But through which complex regulatory mechanisms does vinculin affects MET, activates NK cell toxicity and causes tumor cell immune escape, and through *in vivo* experiments, we confirmed that VCL can affect EMT and tumor immunity by regulating the expression of EPCAM. However, it is but a surface of a larger mechanism that warrants further research. Vinculin has an important relationship with the prognosis and metastasis of gastric cancer patients, and for that, whether we can find its target and allow for precision therapy, is another question that we will have to answer in the future.

## MATERIALS AND METHODS

### Study subjects

All data was downloaded from TCGA website (https://portal.gdc.cancer.gov/) on April 2020, including transcriptome, DNA methylation data, and clinical data of pathologically-diagnosed gastric adenocarcinoma patients, including 416 cases of gastric adenocarcinoma data and corresponding general information. Data without a listed survival time was excluded, final cases included were 416 cases of gastric cancer tissues and 33 cases of surrounding tissues. Inclusion criteria: (a) age of onset ≥ 8 years old; (b) tumor location: stomach; (c) cases confirmed via pathological tests. Exclusion criteria: (a) multigenic tumor; (b) carcinoma in situ; (c) incomplete follow-up data; (d) cases with death within 30 days; (e) cases with known expression levels of VIM, CDH1, S100A4, and EPCAM. See Table 1 for details.

56 cases of gastric cancer was collected from the First Affiliated Hospital of Sun Yat-sen University, 40 of which were male and 16 of which were female. The average age was 58.77±16.07 years, with a range of 34-82 years. This study was approved by the Ethics Committee of the First Affiliated Hospital of Sun Yat-sen University. All samples were obtained with informed consent from the patient. The surgical pathological staging standards met the Chinese national standards (see Table 2).

**Table 2:** Demographics of stomach carcinoma patients of SYSU.

	**High(9)**	**Low(47)**	**P**
male	6	34	0.731
female	3	13	
Age	(51.78 ± 15.13)	(60.10 ± 16.05)	0.158
M staging			
M0	4	39	0.012
M1	5	8	
T staging			
T1	1	11	0.026
T2	0	10	
T3	4	22	
T4	4	4	
N staging			
N0	5	19	0.634
N1	2	7	
N2	1	7	
N3	1	14	
Differentiation			
High	1	2	0.744
Moderately	0	2	
Poorly	8	42	
Undifferentiation	0	1	

Proteomics data of patients with pathologically-diagnosed gastric adenocarcinoma were downloaded from CPTAC website (https://cptac-data-portal.georgetown.edu/study-summary/S025) on April 2020, including data and corresponding general information of 130 cases of gastric adenocarcinoma, which included 130 samples of gastric cancer tissues and its corresponding paracancerous tissues. The data within is displayed by the expression level of cancer tissue minus the expression level of paracancerous tissues. Inclusion criteria: Cases with known expression levels of VIM, CDH1, S100A4 and EPCAM. Due to the limited clinical information of patients in this database, relevant prognostic detail will be temporarily unavailable.

Transcriptomic data of patients with pathologically normal human tissues were downloaded from GTEx website (https://gtexportal.org/home/) in April 2020, which included 9,783 cases of normal human tissues and corresponding general information, which included 621 samples of adipose tissue, 161 samples of adrenal gland, 13 samples of bladder, 595 samples of blood, 753 samples of blood vessel, 102 samples of bone marrow, 1,426 samples of brain, 221 samples of breast, 11 samples of cervix uteri, 384 samples of colon, 805 samples of esophagus, 7 samples of fallopian tube, 493 samples of heart, 38 samples of kidney, 141 samples of liver, 381 samples of lung, 478 samples of muscle, 209 samples of stomach, etc. Inclusion criteria: Cases with known expression levels of VIM, CDH1, S100A4 and EPCAM. Due to the limited clinical information of patients in this database, relevant prognostic detail will be temporarily unavailable.

### RNA extraction and RT-qPCR

Each cancer tissue and its corresponding paracancerous tissues and cells were homogenized with Trizol to extract mRNA and determine its concentration. 5ug of sample RNA, oligo d(T) 1ul, dNTP 1ul, and an appropriate amount of DEPC·H20 was added to a total volume of 12ul and mixed thoroughly. The solution was put in a water bath at 65° C for 10 minutes, then 4uL of 5×first-strand buffer was added, along with 2ul of 1umol/l DTT, 1ul of RNase Inhibitor (RNase In), and 1ul of M-MLV. The solution was then put in a 75° C water bath for 5 min to inactivate the reverse transcriptase. cDNA was amplified using primers. The PCR reaction conditions were: pre-denaturation at 95° C for 5min; followed by denaturation at 60° C for 20s, annealing at 60° C for 20s, and extension at 72° C for 30s, for a total of 40 cycles; then finally at 95° C for 15s. The above reagents and primers were provided by Takara Biomedical Technology Co., Ltd. (Takara, Biotech, Beijing, China). The expression of RNA was detected by the ABI StepOne Real-time PCR System (ABI Biotech, New York, USA). The relative RNA expression was calculated by 2ΔΔct method for GAPDH normalization. (for primer sequence, see Supplementary Table 1).

### Enumeration of hematopoietic cells subsets from gene expression profiles

Using normal gene expression data, the relative proportions of 22 different genotypes were calculated using the CIBERSORT algorithm to infer infiltrating immune cells. For the TCGA data set, ‘voom’ (variance model at the observation-level) was used to convert RNA sequencing data and convert count data to values closer to the microarray results [21]. The 22 cell types inferred by CIBERSORT included B cells, T cells, natural killer cells, macrophages, dendritic cells, eosinophils, neutrophils, and etc. CIBERSORT is a deconvolution algorithm that uses a set of data that is considered to be the smallest expression level of each cell type and represents the data corresponding to the reference gene expression value (547 genes in the "signature matrix"). Based on these algorithms, it could then infer the proportion of cell types in tumor samples [22]. CIBERSORT used Monte Carlo sampling to obtain a p-value for the deconvolution of each sample, thus providing an interval measure of the result. The gene expression data set was written using standard annotation files and data uploaded to the CIBERSORT web portal (http://cibersort.stanford.edu/). The algorithm uses the default signature matrix with 1000 permutations. Heat maps were drawn to illustrate the infiltration of tumor immune cells in different patients, and by analyzing the correlation between immune cells in different mutation groups, correlation heat maps were drawn. In order to explore the differences in infiltrating immune cells in different mutation groups, a violin plot was plotted for visual display.

### WGCNA Co-expression network construction

Gene expression data (mRNA-seq data) were obtained from the TCGA database. A total of 24,991 genes were identified in each sample. An analysis of variance was performed on the results and were sorted from largest to smallest. The SD value of each gene was calculated and sorted from largest to smallest, and then the top 5,000 genes were selected for weighted gene coexpression network analysis (WGCNA).

A gene co-expression network was constructed with the expression data of these 5,000 genes via the WGCNA package in R software [23]. Using the WGCNA adjacency function, an adjacency matrix was constructed by calculating the Pearson correlation between all pairs of genes in the selected sample. In this study, β was used as a soft threshold parameter to ensure a scale-free network. In order to further identify the functional modules in the co-expression network of these 5,000 genes, the adjacency matrix was used to calculate the Topological Overlap Measure (TOM), which represents the overlap in the shared neighborhood.

### Identification of clinical significant modules

Related modules were identified by calculating the correlation between MEs and each phenotype. Then the log10 transformation of the p value (GS = lgP) of the linear regression of gene expression and phenotypic information was defined as gene significance (GS). In addition, module significance (MS) was defined as the average GS of all genes in a module. In general, among all selected modules, the module with the highest absolute value of MS is considered to be the module related to the phenotype.

### PPI network construction of key module gene

The hub gene, which was highly interconnected with the nodes in the module, was considered to have important functions. Top 30 hub genes in the module network was selected as candidate genes for further analysis and verification. The STRING data set is an online biological resource that can decode the interaction between proteins to get the actual, precise functions of the protein [24]. The candidate gene was submitted to the protein interaction of STRING, and the binding confidence interval of the cutoff value is set to 0.4. In Plugin Molecular Complex Detection (MCODE), the significant models with stronger protein-protein connection was calculated and selected, with the default parameters (degree cut ≥ 2, node score cut ≥ 2, K-core ≥ 2, maximum depth = 100). P<0.05 was considered statistically significant.

### Gene ontology and pathway enrichment analysis

The Database for Annotation, Visualization and Integrated Discovery (DAVID) is a database for gene function annotation and visualization [25]. Gene ontology (GO) and Kyoto Encyclopedia of Genes and Genomes (KEGG) uses DAVID (version 6.8; https://david.ncifcrf.gov/) online tools (functional annotation tools) to perform pathway analysis of core genes. The ontology contained three categories: biological processes (BP), cellular components (CC), and molecular functions (MF). According to the adjusted critical standard P < 0.05, Enriched GO terms and KEGG pathways were determined. GSEA v2.0 software(http://software.broadinstitute.org/gsea/index.jsp) was used to perform GSEA analysis [26]. The enrichment score was compared with the enrichment results generated by 1000 random permutations of the genome to obtain the p value and evaluate its statistical significance.

### Survival analysis of hub genes

Online Kaplan-Meier Plotter (http://kmplot.com/analysis/) is a platform containing 10 tumor gene expression information and 1,065 clinical gastric cancer patients' survival data. The website was used to obtain information on core gene expression and patient prognosis, thus aiding in the search of core genes that influence differences in survival [27]. In order to assess the prognostic value of specific genes, patient samples were divided into two groups based on the median gene expression (high expression vs. low expression). Core genes were uploaded to the database to obtain the Kaplan-Meier survival curve, which was used to analyze the overall survival (OS) of gastric cancer patients. 95% confidence interval, p-value and hazard ratio (HR) was calculated and displayed in the graph.

### Preprocessing of DNA methylation chip data

Illumina Infinium HumanMethylation450 BeadChip (Illumina, Inc.) was used to analyze the genome-wide DNA methylation of the data set, and the raw data (the raw data in TCGA) was processed using R package ‘minfi’ version 1.20.0, followed by a background subtraction, quantile normalization and quality control [28]. If the following criteria were met, low-quality probes were excluded: i) undetectable rate of ≥5% of total samples (<0.05); ii) coefficient of variance (<0.05); OR iii Single nucleotide polymorphisms (SNPs) were located in detected CpG dinucleotides) [29]. β-mixture quantile normalization was used to correct type I and type II probe bias [30]. The DNA methylation data of the verification group was standardized [31], and the methylation of core genes was related to the expression data. VanderWeele's mediation analysis was used to study whether the prognostic effects of the seven DNA methylation sites are mediated by affecting the corresponding mRNA expression [32].

### Cell culture and transfection of siRNA

Both MGC-803 and AGS cells were selected from American Type Culture Collection and used within 20 passages. RPMI1640 high glucose medium (Invitrogen) containing 10% calf serum (Gibco) and 100 U/ml penicillin, and 100 mg/ml streptomycin (Invitrogen) was used in an incubator at 37° C and 5% CO_2_, and cells in the logarithmic growth phase were selected for the experiment. 3×10^5^ cells was placed in a 60mm petri dish, and transfected with Lipofectamine 2000, RNAi Max (Invitrogen) and 100nM siRNA 24 hours later. After 24 hours, the transfection mixture was removed and replaced with normal growth medium. Cells were collected at 72h after transfection (Supplementary Figure 11A–11C).

### *In vitro* migration/invasion assays and clone formation assay

The extracellular matrix (ECM) hydrogel was diluted with serum-free RPMl640 at a ratio of 1:1, and 30ul of Transwell polycarbonate membrane was added to each chamber to coat the ECM hydrogel. It was placed in a 37° C incubator for 15 minutes to let it solidify. After 16 hours, AGS cells and MGC-803 cells were digested with 2.5% trypsin, suspended in serum-free medium, and adjusted the cell density to 1×10^5^/mL. 100ul of single cell suspension was added to the upper chamber of each chamber, and 600ul of culture medium containing 10% FBS was added to the lower chamber of the 24-well plate in the chamber, and it was set in a 37° C incubator for routine cultivation for 24 hours and 36 hours; each group containing a 3-cell parallel cell-culture chamber, and the experiment was repeated 3 times. After incubation, the chamber was removed, and the matrigel and cells in the upper chamber of the cell were gently wipe off, stained with 0.1% crystal violet for 5 minutes, the excess dye was rinsed off with water; the cells passed through the membrane were counted under a 200X microscope and taken photos of. 5 fields of view were randomly selected at the top, bottom, center, left, and right, and the average value was taken. Cell migration assay: a Transwell chamber that was not coated with ECM hydrogel was used, with 100-cell suspension added to the upper chamber of the chamber, and the rest is identical as in the invasion assay.

The cell suspension was diluted, and each group of cells was respectively seeded in a six-well plate containing 37° C pre-warmed culture medium at a gradient density of 200 cells per dish, and gently rotated to ensure even dispersion of the cells. It was placed in a cell incubator at 37° C, 5% CO_2_ and saturated humidity for 2 to 3 weeks. The plate was turned upside down and superimposed onto a transparent film with a grid, and the clones were counted by the naked eye, or counted on a low power microscope, each clone containing more than 10 cells. Finally, the clone formation rate was calculated.

### Western blotting

The total protein of the extracted cells was lysed in frozen cell lysis buffer (NEB) containing 1 mM PMSF and 1:100 protease inhibitor cocktail (Sigma). The lysate was pre-cleared with 15×l protein A Sepharose 4B beads (Sigma) at 4° C for 30 minutes. NE-PER™ Nuclear and Cytoplasmic Extraction Reagents (Thermo Fisher, 78833) were used to extract the nucleoprotein of the cell. The pre-determined BCA (Pierce, Rockford, IL) and Western Blot procedures were as described in the literature. (Vinculin Antibody, Rabbit Polyclonal, Affinity Biosciences Cat# AF5122, RRID:AB_2837608, EPCAM Antibody, Rabbit Polyclonal, Affinity Biosciences Cat# 21050-1-AP)

### Immunofluorescent staining

Immunofluorescence staining of strain: the anti-body was placed in a glass bottom tissue culture plate (ibidi), fixed with 5% NBF for 10 min, and then blocked with PBS containing 10% FCS, 1% BSA (Sigma) and 0.2% Triton-X. The primary antibody was incubated in blocking buffer at 4° C for 16h. The fluorescent secondary antibody was incubated with 3 DAPI in blocking buffer at 20° C for 1-6h. Fluorescence staining was imaged on a Zeiss LSM 780 confocal microscope. (Vinculin Antibody, Rabbit Polyclonal, Affinity Biosciences Cat# AF5122, RRID:AB_2837608, EPCAM Antibody, Rabbit Polyclonal, Affinity Biosciences Cat# 21050-1-AP)

### Immunohistochemical analysis

A total of 56 archived gastric cancer tissues were fixed with 4% paraformaldehyde, then paraffin-embedded, sectioned (5mm), deparaffinized, rehydrated, and antigen extracted. The tissue part was used to incubate the CAS blocking buffer and subsequently used to incubate the main antibody (E-Cadherin Antibody, Rabbit Polyclonal, PROTEINTECH, Catalog number: 20874-1-AP. Vimentin Antibody, Rabbit Polyclonal, PROTEINTECH, Catalog number: 10366-1-AP. Vinculin Antibody, Rabbit Polyclonal, Affinity Biosciences Cat# AF5122, RRID:AB_2837608). It was then overnighted at 4° C and DAKO envisio-hrp (DAKO) was used to check the reactivity afterward. By multiplying the percentage of positive cells (P) by the intensity (I), the degree of immunostaining is scored according to the following formula: H = P × I. The range of P is 0 to 4 (5% scored 0; 5-25% scored 1; 25–50% scored 2; 50–75% scored 3; 75–100% scored 4). The range of I is 0 to 3 (0, no staining; 1, weak; 2, medium; 3, strong). The staining index score was calculated by multiplying P by I, and the resulting range is 0-12. The staining index score is defined as followed: 0-3 as negative; 4-12 as positive.

### Statistical analyses

Pearson’s χ^2^ test was used to measure the differences in mRNA and IHC expression between the gastric cancer tissues. The overall survival (OS) was calculated by survival analysis. OS was defined as the time from the initial diagnosis of primary gastric cancer to death from any cause. The survival rate was analyzed using Kaplan-Meier’s, and the difference in survival curve was analyzed by a log-rank test. Spearman’s rank correlation (rs) was used to explore the relationship between infiltrating immune cells in tumor and DNA methylation and RNA expression between different mutation groups. All statistical analyses were performed using IBM SPSS statistical software version 20.0 (IBM Corp., Armonk, NY, USA) and R version 3.3.0 (The R Foundation). The P value is two-sided, and P <0.05 was used as indication that the difference is statistically significant.

## Supplementary Material

Supplementary Figures

Supplementary Tables
