# Supplementary material for: High expression of vinculin predicts poor prognosis and distant metastasis and associates with influencing tumor-associated NK cell infiltration and epithelial-mesenchymal transition in gastric cancer
Source: Aging (Albany NY). 2021 Feb 1;13(4):5197–225. doi: 10.18632/aging.202440 (PMC7950221; doi:10.18632/aging.202440)
Supplement: Supplementary Figures [file aging-13-202440-s001.pdf]

SUPPLEMENTARY FIGURES

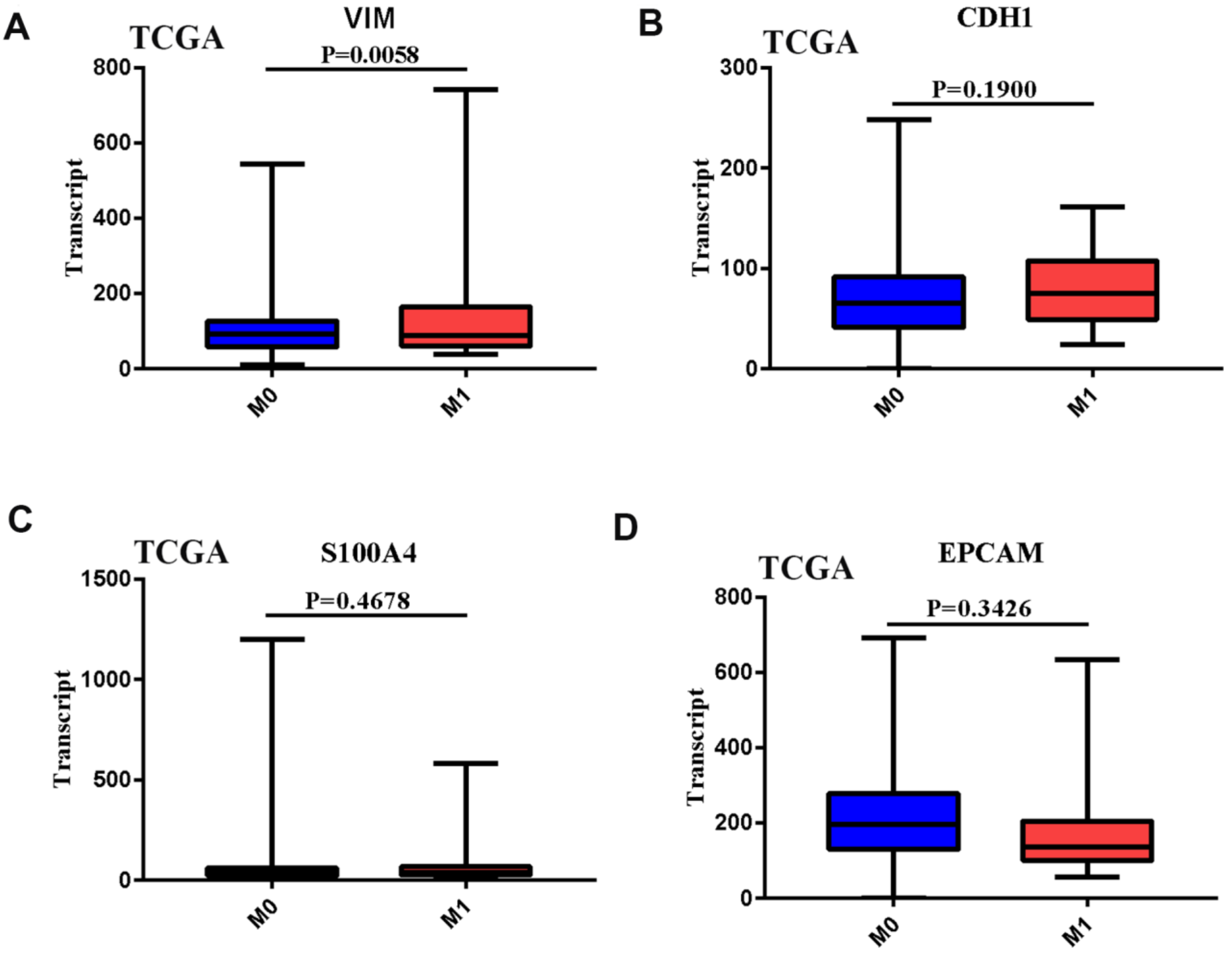

Supplementary Figure 1. (**A**–**D**) VIM, CDH1, S100A4, EPCAM mRNA expression in GC tissues of TCGA Cohort patient of Distant metastases**.**

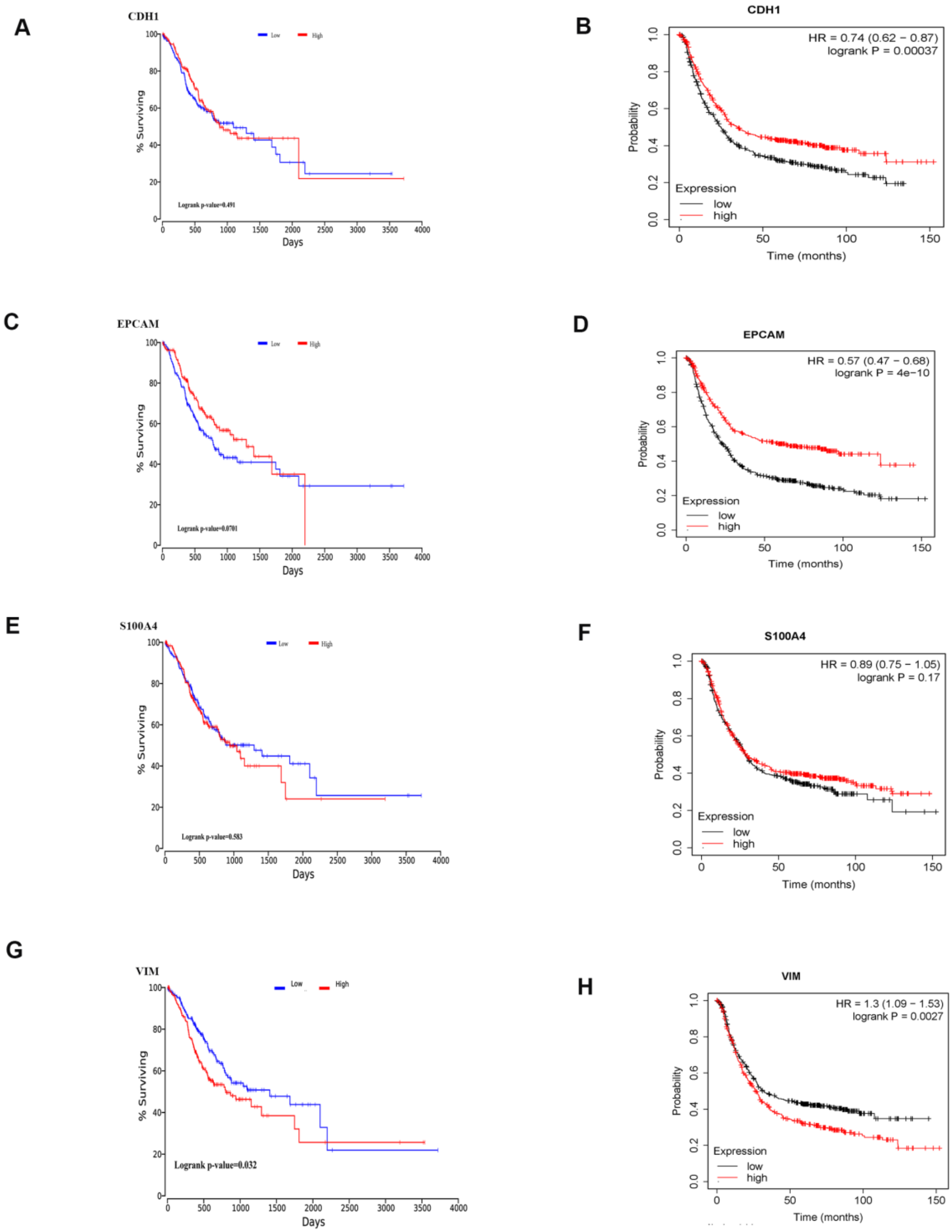

Supplementary Figure 2. (**A**) Kaplan-Meier survival curves of overall survival in TCGA GC patients based on VIM mRNA expression. Log-rank test was used to compare differences between two groups. (**B**) Kaplan-Meier survival curves of overall survival in GEO GC patients based on VIM mRNA expression. Log-rank test was used to compare differences between two groups. (**C**) Kaplan-Meier survival curves of overall survival in TCGA GC patients based on CDH1 mRNA expression. Log-rank test was used to compare differences between two groups. (**D**) Kaplan-Meier survival curves of overall survival in GEO GC patients based on CDH1 mRNA expression. Log-rank test was used to compare differences between two groups. (**E**) Kaplan-Meier survival curves of overall survival in TCGA GC patients based on S100A4 mRNA expression. Log-rank test was used to compare differences between two groups. (**F**) Kaplan-Meier survival curves of overall survival in GEO GC patients based on S100A4 mRNA expression. Log-rank test was used to compare differences between two groups. (**G**) Kaplan-Meier survival curves of overall survival in TCGA GC patients based on EPCAM mRNA expression. Log-rank test was used to compare differences between two groups. (**H**) Kaplan-Meier survival curves of overall survival in GEO GC patients based on EPCAM mRNA expression. Log-rank test was used to compare differences between two groups.

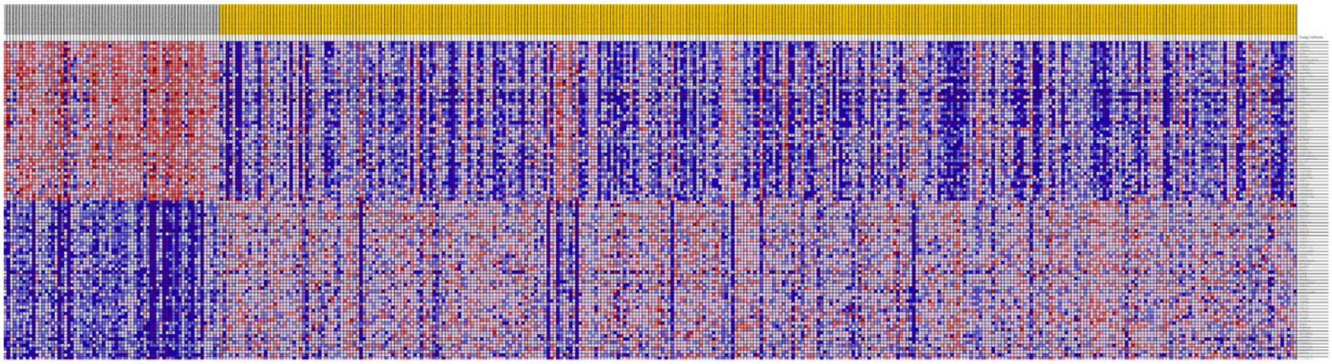

Supplementary Figure 3. Heat map showing the up- and downregulated genes in EMT. The horizontal axis represents the sample name. Gray indicates EMT patients, and yellow indicates non-EMT. Red represents upregulated genes, and blue represents downregulated genes.

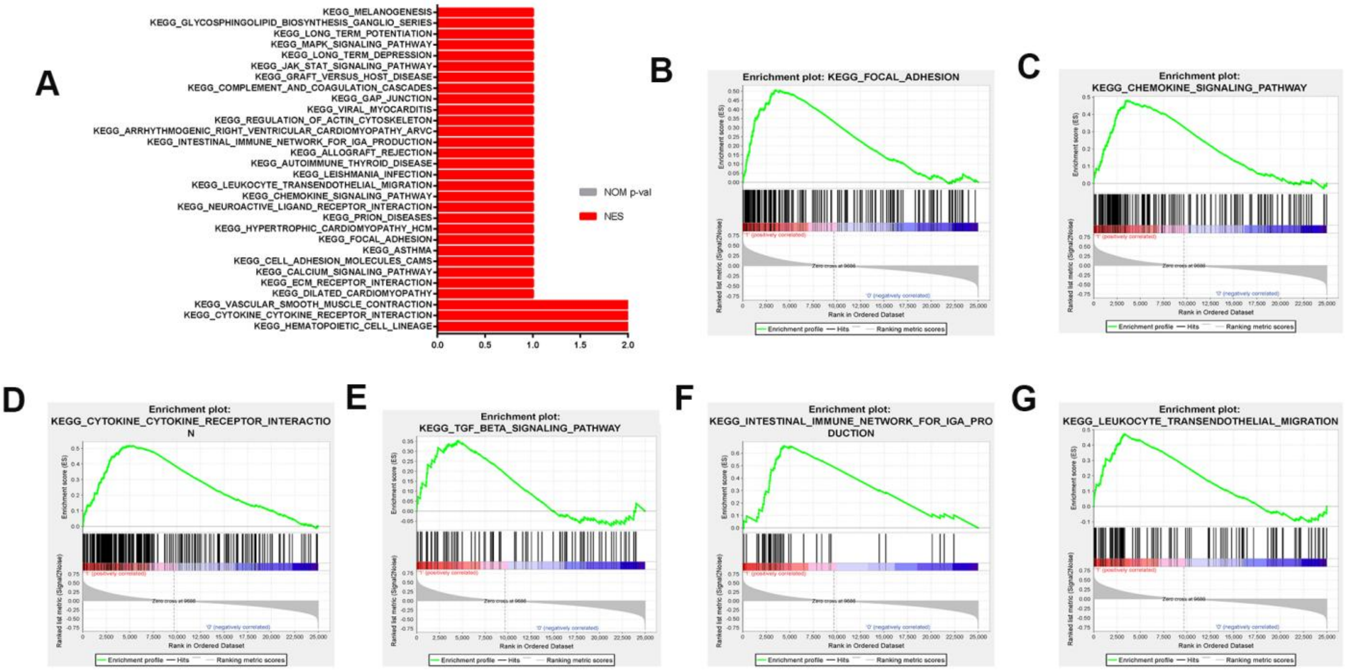

Supplementary Figure 4. (**A**) Functional enrichment and pathway analysis of the EMT. (**B**) Focal adhesion pathway in EMT. (**C**) Chemokine signaling pathway in EMT. (**D**) Cytokine cytokine receptor Interaction pathway in EMT. (**E**) TGF beta signaling pathway in EMT. (**F**) Intestinal immune network for IGA production pathway in EMT. (**G**) Leukocyte transendothelial migration pathway in EMT.

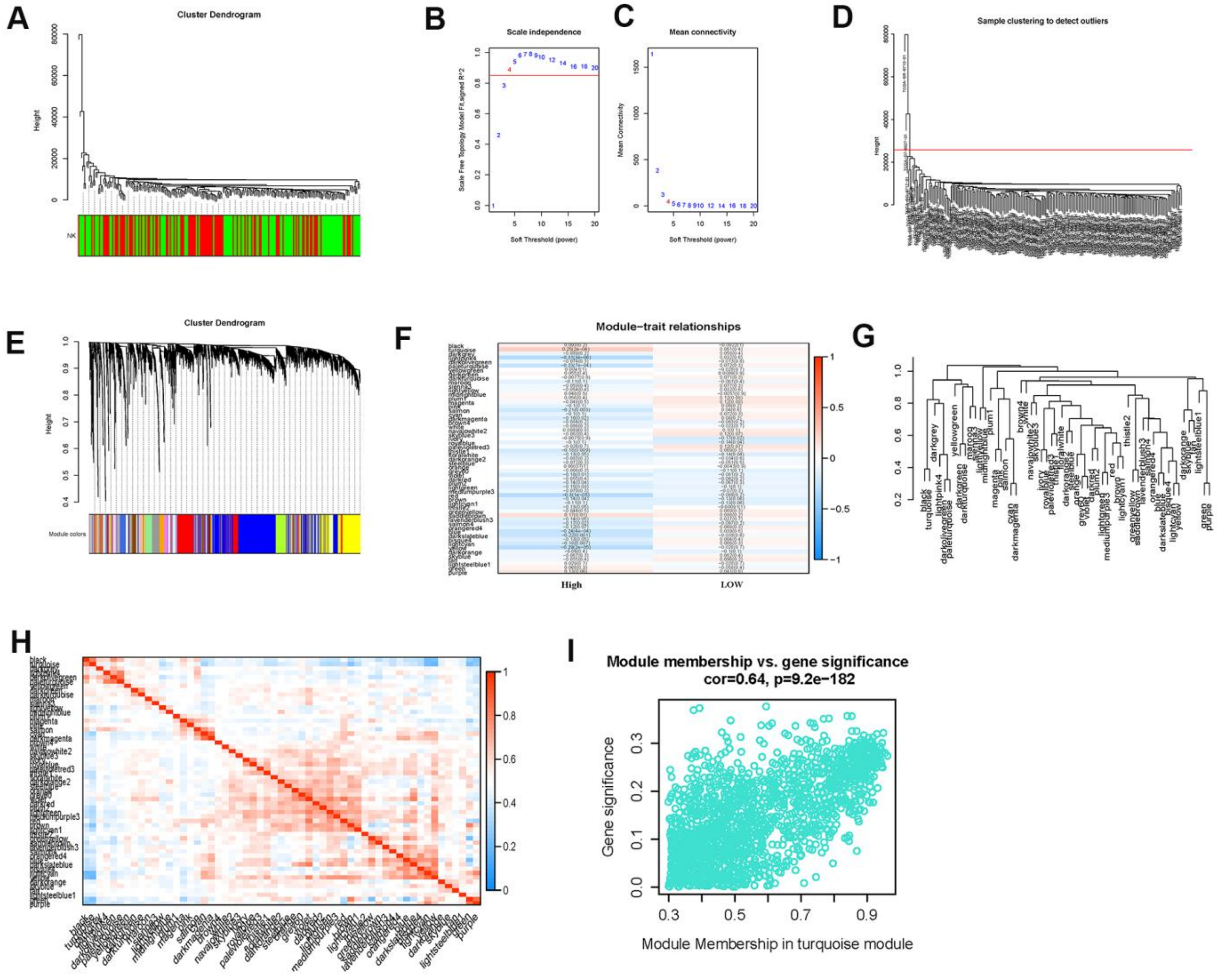

Supplementary Figure 5. Clustering of samples and determination of soft-thresholding power. Clustering of samples and determination of soft-thresholding power. (**A**) The clustering was based on the expression data of TCGA. The top 5,000 genes with the highest SD values were used for the analysis by WGCNA. The color intensity was proportional to expression status (NK cell low, and NK cell high). (**B**) analysis of the scale-free fit index for various soft-thresholding powers (β). (**C**) Analysis of the mean connectivity for various soft-thresholding powers. In all, 4 was the most fit power value. (**D**) The cluster dendrogram of module eigengenes. (**E**) The cluster dendrogram of genes in TCGA. Each branch in the figure represents one gene, and every color below represents one co-expression module. (**F**) Heat map of the correlation between module eigengenes and the status of NK cell. (**G**) The Turquoise module was the most positively correlated with NK cell high infiltration. (**H**) heat map plot of the adjacencies in the hub gene network. (**I**) Scatter plot of module eigengenes in the Turquoise module.

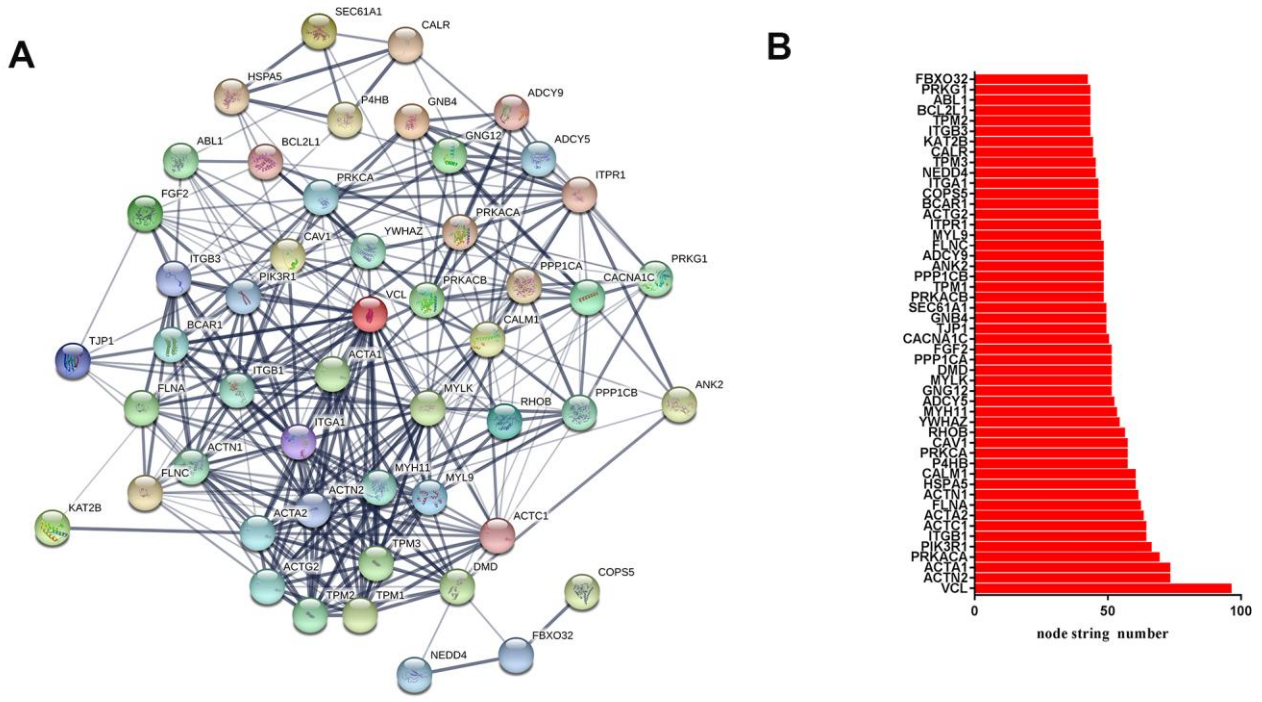

Supplementary Figure 6. (**A**) The top hub genes in the Turquoise module. (**B**) The number of node string of hub genes in the Turquoise module. Edges represent protein-protein associations. Cambridge blue: from curated databases. Violet: experimentally determined. Green gene: neighborhood. Red: gene fusions. Blue: gene co-occurrence. Reseda: textmining. Black: co-expression. Lila : protein homology.

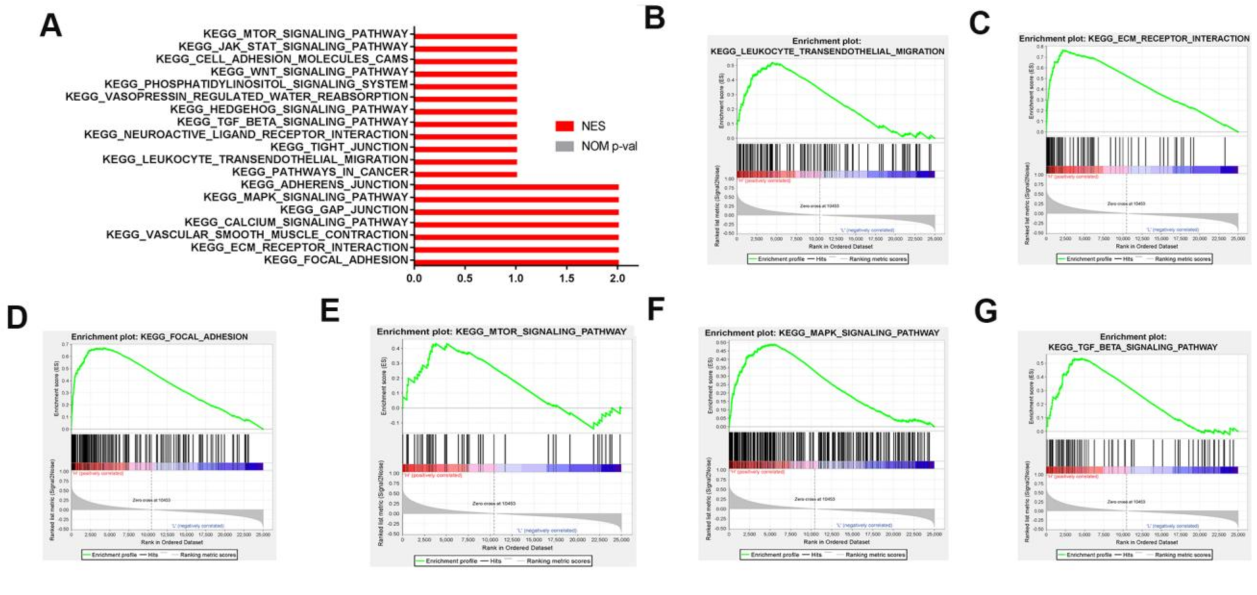

Supplementary Figure 7. (**A**) Functional enrichment and pathway analysis of the VCL expression. (**B**) Leukocyte trans-endothelial migration pathway in VCL high expression. (**C**) ECM receptor interaction pathway in VCL high expression. (**D**) Focal adhesion pathway in VCL high expression. (**E**) MTOR signaling pathway in VCL high expression. (**F**) MAPK signaling pathway in VCL high expression. (**G**) TGF beta signaling pathway in VCL high expression.

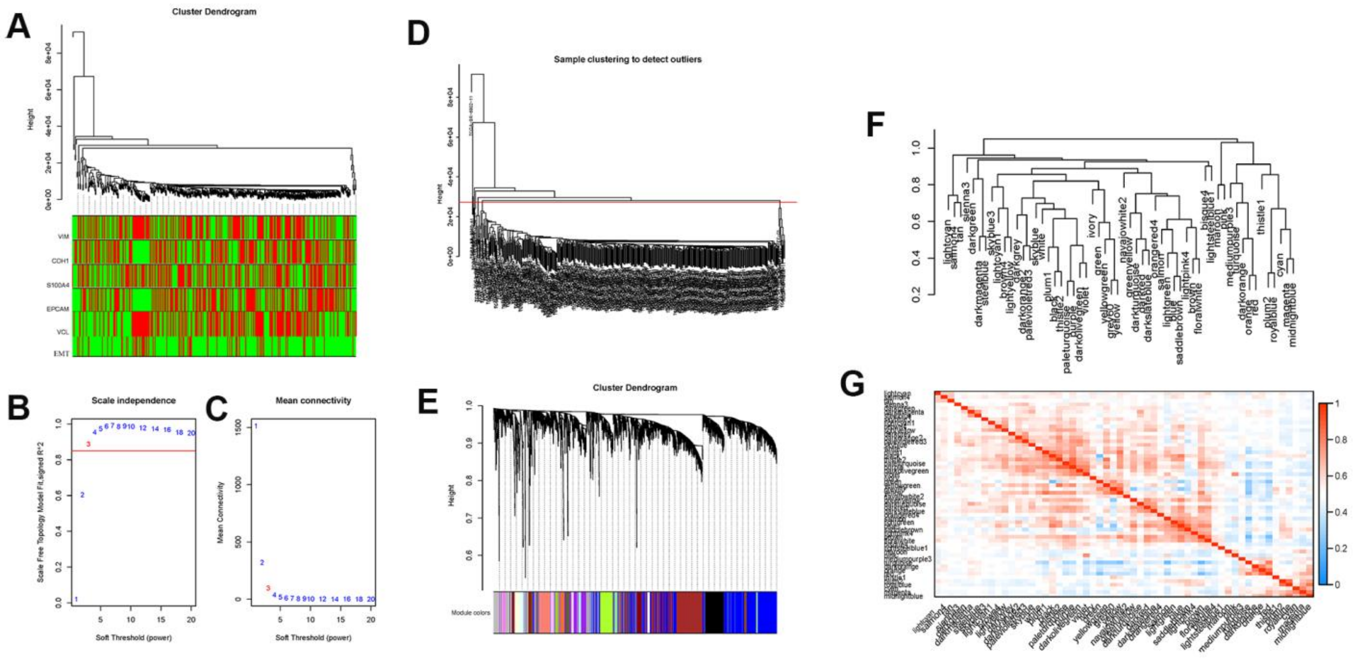

Supplementary Figure 8. Clustering of samples and determination of soft-thresholding power with VIM, CDH1, S100A4, EPCAM, VCL expression and EMT. (**A**) The clustering was based on the expression data of TCGA. The top 5,000 genes with the highest SD values were used for the analysis by WGCNA. The color intensity was proportional to its status (VIM, CDH1, S100A4, EPCAM, VCL expression and EMT). (**B**) Analysis of the scale-free fit index for various soft-thresholding powers (β). (**C**) Analysis of the mean connectivity for various soft-thresholding powers. In all, 3 was the most fit power value. (**D**) The cluster dendrogram of module eigengenes. (**E**) The cluster dendrogram of genes in TCGA. Each branch in the figure represents one gene, and every color below represents one co-expression module. (**F**) Hierarchical clustering of module hub genes that summarize the modules yielded in the clustering analysis. (**G**) Heat map plot of the adjacencies in the hub gene network.

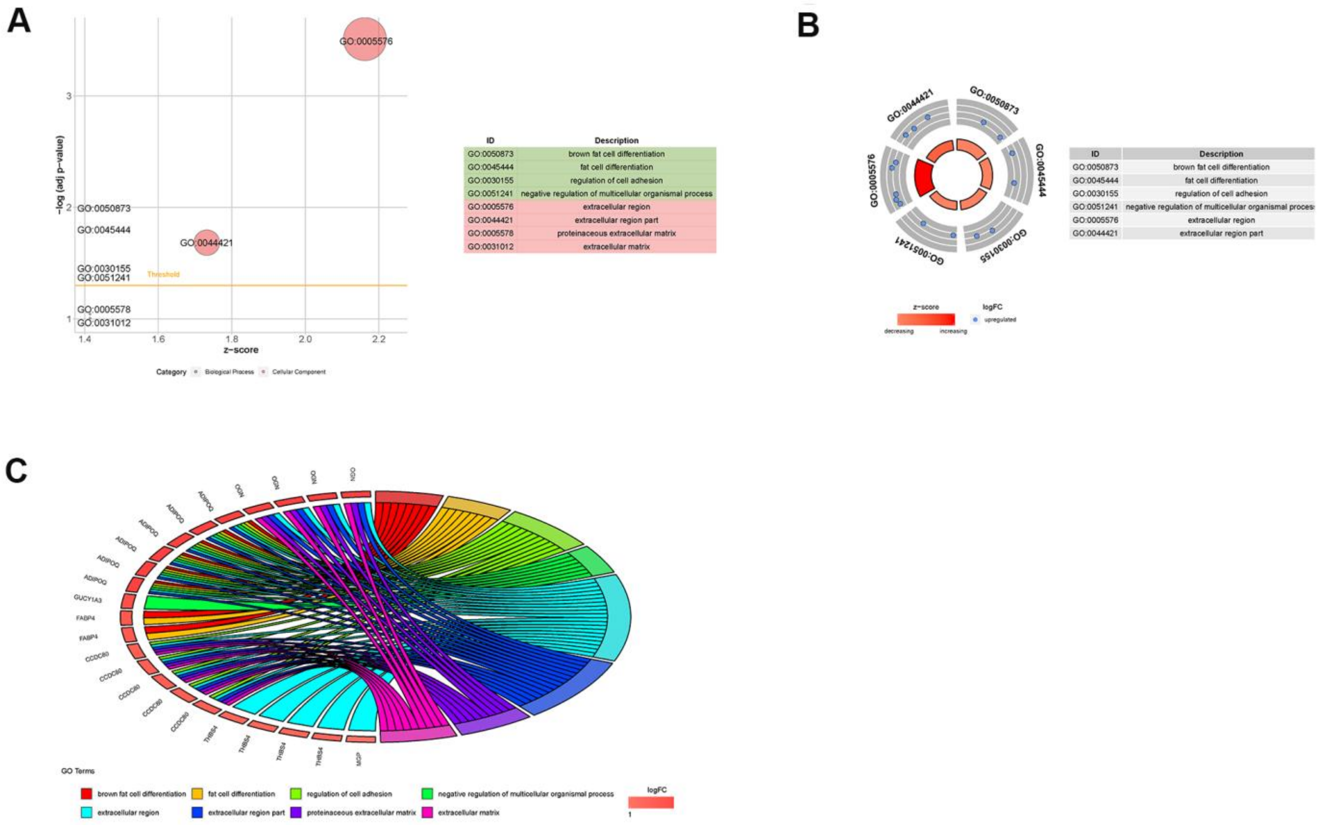

Supplementary Figure 9. Functional enrichment and pathway analysis of the differentially expressed genes in red module. GO, Gene Ontology. (**A**) The z-score of the enrichment pathway and the change of P value. (**B**) Significantly different enrichment pathways. (**C**) The relationship between enrichment pathways and genes.

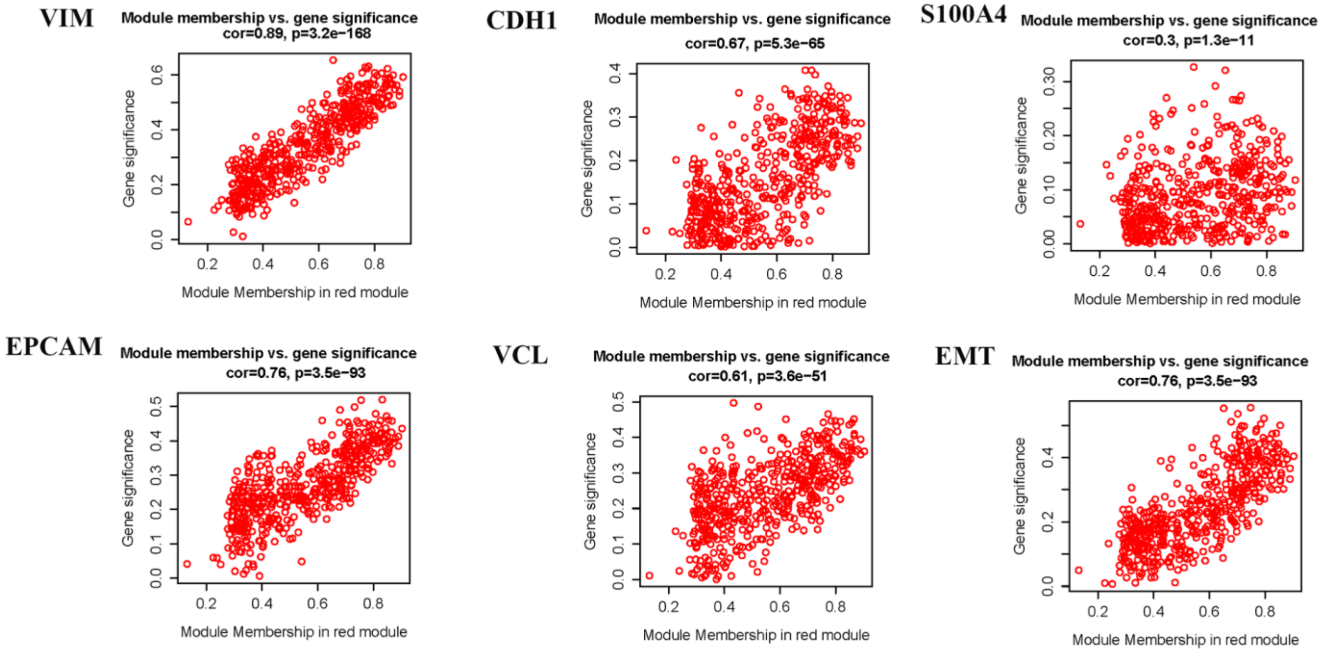

Supplementary Figure 10. Scatter plot of module eigengenes in the red module of VIM, CDH1, S100A4, EPCAM, VCL expression and EMT.

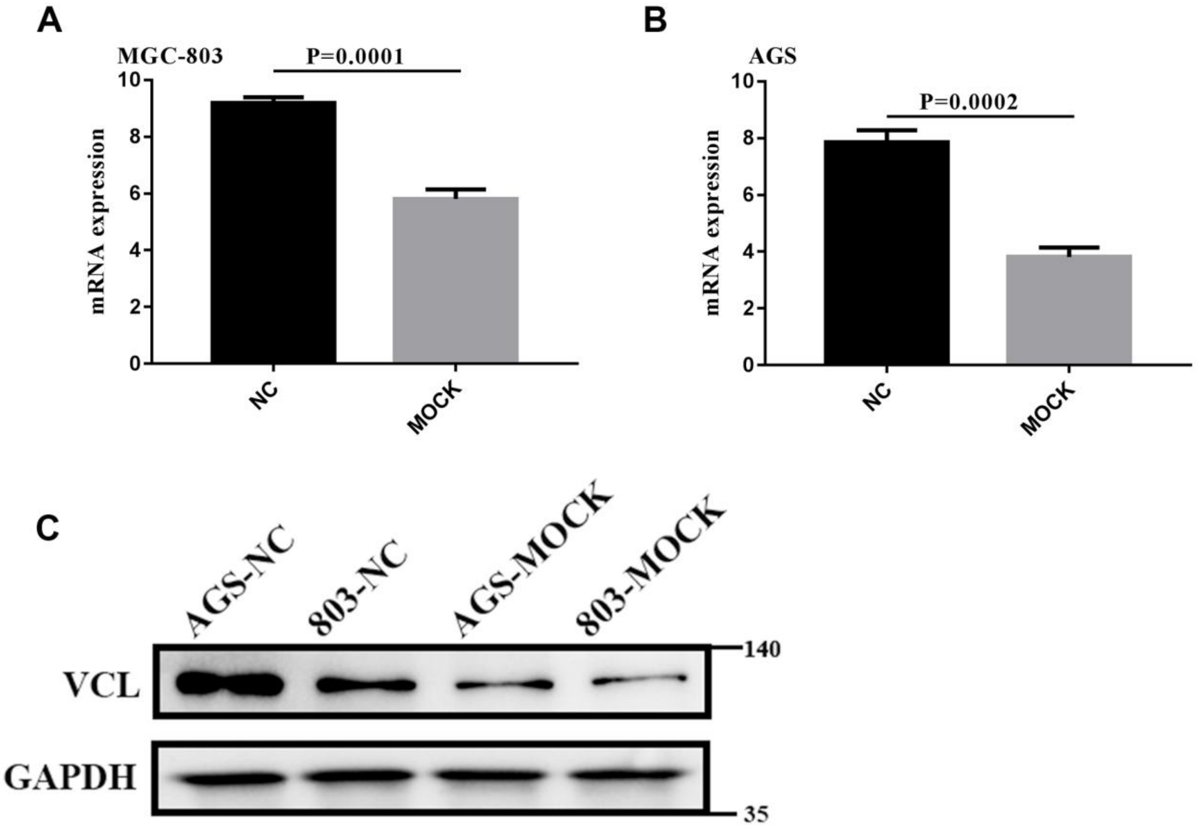

Supplementary Figure 11. (**A**, **B**) The VCL RNA expression of VCL silent group and blank group. (**C**) The VCL expression of VCL silent group and blank group.
