# Supplementary material for: High expression of vinculin predicts poor prognosis and distant metastasis and associates with influencing tumor-associated NK cell infiltration and epithelial-mesenchymal transition in gastric cancer
Source: Aging (Albany NY). 2021 Feb 1;13(4):5197–225. doi: 10.18632/aging.202440 (PMC7950221; doi:10.18632/aging.202440)
Supplement: Supplementary Tables [file aging-13-202440-s002.pdf]

SUPPLEMENTARY TABLES

Supplementary Table 1. mRNA primer sequence.

Primer name	Primer sequence
VIM	F:5′-AGGCAAAGCAGGAGTCCACTGA-3′
	R:5′-ATCTGGCGTTCCAGGGACTCAT-3′
CDH1	F:5′-GCCTCCTGAAAAGAGAGTGGAAG-3′
	R:5′-TGGCAGTGTCTCTCCAAATCCG-3′
S100A4	F:5′-CAGAACTAAAGGAGCTGCTGACC-3′
	R:5′-CTTGGAAGTCCACCTCGTTGTC-3′
EPCAM	F:5′-GCCAGTGTACTTCAGTTGGTGC-3′
	R:5′-CCCTTCAGGTTTTGCTCTTCTCC-3′
VCL	F:5′-TGAGCAAGCACAGCGGTGGATT-3′
	R:5′-TCGGTCACACTTGGCGAGAAGA-3′
GAPDH	F:5′-GTCTCCTCTGACTTCAACAGCG-3′
	R:5′-ACCACCCTGTTGCTGTAGCCAA-3′

Supplementary Table 2. Gene statistics in each module of NK status.

Module	Genes	Module	Genes	Module	Genes	Module	Genes
bisque4	42	floralwhite	45	navajowhite2	38	steelblue	79
black	481	green	581	orange	90	tan	208
blue	1554	greenyellow	218	orangered4	53	thistle1	39
brown	824	grey	1250	paleturquoise	77	thistle2	39
brown4	44	grey60	143	palevioletred3	38	turquoise	1571
cyan	150	ivory	46	pink	298	violet	75
darkgreen	109	lavenderblush3	34	plum1	64	white	86
darkgrey	91	lightcyan	144	plum2	41	yellow	754
darkmagenta	72	lightcyan1	47	purple	275	yellowgreen	68
darkolivegreen	74	lightgreen	139	red	572		
darkorange	89	lightpink4	36	royalblue	125		
darkorange2	44	lightsteelblue1	49	saddlebrown	80		
darkred	116	lightyellow	138	salmon	169		
darkslateblue	41	magenta	296	salmon4	39		
darkturquoise	91	maroon	37	sienna3	68		
bisque4	42	mediumpurple3	50	skyblue	81		
black	481	midnightblue	149	skyblue3	64		

Supplementary Table 3. Gene statistics in each module of EMT status.

Module	Genes	Module	Genes	Module	Genes	Module	Genes
bisque4	42	greenyellow	218	orangered4	53	thistle1	39
black	481	grey	1250	paleturquoise	77	thistle2	39
blue	1554	grey60	143	palevioletred3	38	turquoise	1571
brown	824	ivory	46	pink	298	violet	75
brown4	44	lavenderblush3	34	plum1	64	white	86
cyan	150	lightcyan	144	plum2	41	yellow	754
darkgreen	109	lightcyan1	47	purple	275	yellowgreen	68
darkgrey	91	lightgreen	139	red	572		
darkmagenta	72	lightpink4	36	royalblue	125		
darkolivegreen	74	lightsteelblue1	49	saddlebrown	80		
darkorange	89	lightyellow	138	salmon	169		
darkorange2	44	magenta	296	salmon4	39		
darkred	116	maroon	37	sienna3	68		
darkslateblue	41	mediumpurple3	50	skyblue	81		
darkturquoise	91	midnightblue	149	skyblue3	64		
floralwhite	45	navajowhite2	38	steelblue	79		
green	581	orange	90	tan	208		
